# Survival after live donor *versus* deceased donor liver transplantation: propensity score–matched study

**DOI:** 10.1093/bjsopen/zrae058

**Published:** 2024-06-05

**Authors:** Christof Kaltenmeier, Hao Liu, Xingyu Zhang, Armando Ganoza, Andrew Crane, Colin Powers, Vikraman Gunabushanam, Jaideep Behari, Michele Molinari

**Affiliations:** Department of Surgery, University of Pittsburgh Medical Center, Pittsburgh, Pennsylvania, USA; Department of Surgery, University of Pittsburgh Medical Center, Pittsburgh, Pennsylvania, USA; School of Health and Rehabilitation Sciences, University of Pittsburgh, Pittsburgh, Pennsylvania, USA; Department of Surgery, University of Pittsburgh Medical Center, Pittsburgh, Pennsylvania, USA; Department of Surgery, University of Pittsburgh Medical Center, Pittsburgh, Pennsylvania, USA; Department of Surgery, University of Pittsburgh Medical Center, Pittsburgh, Pennsylvania, USA; Department of Surgery, University of Pittsburgh Medical Center, Pittsburgh, Pennsylvania, USA; Department of Medicine, University of Pittsburgh Medical Center, Pittsburgh, Pennsylvania, USA; Department of Surgery, University of Pittsburgh Medical Center, Pittsburgh, Pennsylvania, USA

## Abstract

**Background:**

For individuals with advanced liver disease, equipoise in outcomes between live donor liver transplant (LDLT) and deceased donor liver transplant (DDLT) is uncertain.

**Methods:**

A retrospective cohort study was performed using data extracted from the Scientific Registry of Transplant Recipients. Adults who underwent first-time DDLT or LTDL in the United States between 2002 and 2020 were paired using propensity-score matching with 1:10 ratio without replacement. Patient and graft survival were compared using the model for end-stage liver disease (MELD) score for stratification.

**Results:**

After propensity-score matching, 31 522 DDLT and 3854 LDLT recipients were included. For recipients with MELD scores ≤15, LDLT was associated with superior patient survival (HR = 0.92; 95% c.i. 0.76 to 0.96; *P* = 0.013). No significant differences in patient survival were observed for MELD scores between 16 and 30. Conversely, for patients with MELD scores >30, LDLT was associated with higher mortality (HR 2.57; 95% c.i. 1.35 to 4.62; *P* = 0.003). Graft survival was comparable between the two groups for MELD ≤15 and for MELD between 21 and 30. However, for MELD between 16 and 20 (HR = 1.15; 95% c.i. 1.00 to 1.33; *P* = 0.04) and MELD > 30 (HR = 2.85; 95% c.i. 1.65 to 4.91; *P* = 0.001), graft survival was considerably shorter after LDLT. Regardless of MELD scores, re-transplantation rate within the first year was significantly higher after LDLT.

**Conclusions:**

In this large propensity score–matched study using national data, comparable patient survival was found between LDLT and DDLT in recipients with MELD scores between 16 and 30. Conversely, for patients with MELD > 30, LDLT was associated with worse outcomes. These findings underscore the importance of transplant selection for patients with high MELD scores.

## Teaser

We compared the outcomes of adults who underwent either LDLT or DDLT in the United States using propensity-score matching stratified by MELD score.

Our main finding was that recipients of LDLT with MELD scores exceeding 30 experienced significantly worse outcomes than DDLT recipients. Comparable patient survival was found between the two groups when patients underwent surgery with MELD scores ranging from 16 to 30.

For patients with MELD equal to or less than 15, LDLT was associated with better survival.

The risk of graft failure and the need for re-transplantation were significantly higher after LDLT across all the MELD strata.

## Introduction

Live donor liver transplantation (LDLT) has emerged as an effective strategy to expand the pool of available liver grafts (LT)^1–3^. LDLT is believed to be associated with higher survival rates and lower healthcare costs in comparison to deceased donor liver transplantation (DDLT)^4^. These advantages, however, are counterbalanced by the technical complexity of the procedure as well as the involvement of healthy individuals who must undergo major surgeries and bear the risk of serious adverse events, income loss, and potential long-term sequelae necessitating further interventions^5^.

In Western countries where organ donation from deceased donors is common, LDLTs are usually performed for patients with non-competitive model for end-stage liver disease (MELD) scores as, despite their low probability of receiving organ offers, they are at risk of developing life-threatening complications while on the waiting list^1,3,6–8^. Historically, the selection of patients with low MELD scores for LDLT was influenced by early research suggesting inferior outcomes in comparison to DDLT in the presence of severe liver decompensation^9,10^. Another rationale is that because the MELD score is the benchmark for the allocation of organs from deceased donors, patients with high MELD scores have a substantial probability of receiving DDLTs. Therefore, organ allocation policies in countries where donation after death is socially acceptable mitigate the necessity of LDLT for high MELD score patients^11^. Over the past few decades, however, significant advancements in surgical expertise and perioperative care have made LDLT a safe procedure for both donors and recipients^3,4^. Consequently, to reduce the risk of death on the waiting list due to the persistent gap between the number of donors and the number of potential recipients, there has been growing interest in expanding the use of LDLT irrespective of recipients’ MELD scores even in countries where deceased organ donation is common^8^.

Although recent research has shown that LDLT is an excellent option for patients with MELD scores as low as 11^3^, it is unclear whether there is equipoise between LDLT and DDLT for individuals with more advanced liver disease. Previous studies have compared the outcomes of LDLT with the outcomes of DDLT recipients without adjusting for the significant differences between the two groups and concluded that LDLT provides a superior survival and is more cost-effective than DDLT^4^. Yet, this conclusion is deceptive because in the United States, LDLT is usually performed before patients develop significant liver decompensation. To address the lack of studies comparing LDLT to DDLT in recipients with similar characteristics, we conducted a stepwise study using propensity-score matching to pair consecutive adults who underwent LDLT or DDLT between 2002 and 2020 in the United States. Our primary aim was to compare patient survival stratified by MELD scores at the time of surgery^3,4^. Our secondary aim was to test whether there is equipoise for graft survival between the two treatments.

## Patients and methods

### Data source and study design

This retrospective case–control study used data extracted from the Scientific Registry of Transplant Recipients (SRTR), a national data set that collects information on all donors, wait-listed candidates and transplant recipients in the United States, submitted by the members of the Organ Procurement and Transplant Network.

### Study population

The study population included patients aged 18 years or older who underwent DDLT or LDLT between 1 January 2002 and 31 December 2020. Patients who underwent redo LT, multi-visceral transplant recipients, patients who received incompatible blood group ABO grafts and patients with hepatic malignancies other than hepatocellular carcinoma were excluded. Last follow-up was set as 31 December 2021.

### Primary and secondary outcomes

The primary outcome was patient survival, and the secondary outcome was graft survival. Patient survival was defined as the interval between the date of liver transplantation and the date of death, from any cause. Graft survival was defined as the interval between the date of liver transplantation and the date of death, of re-listing or re-transplantation, whichever came first. Death-censored graft failure was defined as the period between the date of surgery and the date of re-listing or re-transplantation with censoring used for patients who died with functioning grafts.

### Stratification

Patients were stratified based on the MELD score obtained at the time of LT. Comparisons between the two groups were performed before and after propensity-score matching. The biochemical MELD without exception points was used to stratify the cohort using increments of five points starting from a baseline MELD of 15. Patients with MELD ≤ 15 represented the reference stratum. Other strata were patients with MELD = 16–20, MELD = 21–25, MELD = 26–30 and MELD > 30. MELD 15 was selected as the starting point for the stratification of the study population based on the results of a landmark study by Merion *et al*.^12^ indicating that, for patients with MELD scores equal or above 15, LT provides a survival advantage in comparison to best supportive care.

### Variables

Data analysed in this study included recipient, donor and operative characteristics. Recipients’ parameters were age at transplantation, sex, the primary cause of liver disease, MELD score^9^, BMI^13^, history of diabetes, need for dialysis, history of portal vein thrombosis, history of trans-hepatic portosystemic shunt (TIPSS), history of spontaneous bacterial peritonitis, history of previous abdominal surgeries, functional status measured using the Karnofsky performance scale^14^, need for ventilator support or life support before surgery and socioeconomic variables. Socioeconomic variables included recipients’ highest degree of education, type of healthcare insurance (private, public or other) and race/ethnicity. Donors’ characteristics included age, sex and BMI. Operative variables included cold ischaemia time (CIT).

### Definitions

Patient and donor ethnicity/race were categorized using the same definitions reported in SRTR. The ethnic/racial groups were White/Caucasian, Black/African American, Hispanic/Latino, Asian/Pacific Islanders and others. The primary source of payment for LT was categorized into private insurance, Medicare/Medicaid, other public insurance and others. The recipient's highest degree of education was categorized into grades 1–8 (Elementary–Middle School), grades 9–12 (High School), College/University or more advanced studies. Waiting time was measured from the date of listing to the date of surgery, regardless of the length of time candidates spent as inactive on the waiting list. For DDLT recipients, CIT was calculated as the time between the cross-clamp of the donor aorta and the time when the liver graft was removed from the cold storage unit during the index operation. For LDLT, ischaemia time was defined as the difference between the time when the hepatic artery and portal vein were cross-clamped and the time when the liver graft was removed from the cold preservation solution.

### Statistical analysis

The mean and s.d. were used to present normally distributed continuous variables and the median and i.q.r. were used for non-parametric continuous variables. Categorical variables were presented using frequencies and percentages. For summary statistics, the Student *t*-test, analysis of variance, χ^2^ and Mann–Whitney *U* tests were used as appropriate. Survival functions were generated using the Kaplan–Meier method^15^ and the log-rank test was used for comparisons between the two groups. Patients who were alive at the last follow-up or lost at the follow-up were censored. For graft survival, censoring occurred when grafts were still functioning at the last follow-up. Cox regression models were used to identify relevant predictors for survival.

### Propensity-score matching

Propensity-score matching was used to pair LDLT and DDLT recipients using the following variables: sex, age at transplantation, race-ethnicity, BMI, level of education, functional status, type of healthcare insurance, blood group type, the primary cause of liver disease, history of diabetes, need for dialysis before LT, need for ventilator support, history of bacterial peritonitis, history of TIPSS, history of portal vein thrombosis, history of previous abdominal surgeries, need for life support, donor sex, donor BMI, donor age and year of transplantation.

Propensity-score matching was set with a 1:10 ratio between LDLT and DDLT recipients without replacement^16^. A nearest-neighbour matching algorithm with callipers of 0.01 was used to pair LDLT and DDLT patients belonging to the same MELD stratum. The standardized mean difference between the parameters used for the propensity-score matching was compared before and after propensity-score matching. Because CIT was unequally distributed between the two groups, it could not be used to pair LDLT with DDLT recipients. Therefore, we assessed whether differences in CIT played an important role in the findings of this study by performing sensitivity analyses using the median CIT of DDLT recipients.

IBM SPSS Statistics software for MacIntosh (IBM Corporation, Armonk, NY, United States, released 2021, Version 28) was used for all statistical analyses. All *P* were two-sided, and *P* < 0.05 were considered statistically significant and no imputations of missing data were used.

### Reporting and ethics

The reporting of this study follows the guidelines described in the STROBE statement^17^. All the procedures and methods for the conduction of this study complied with the Declaration of Helsinki on ethical principles for medical research involving human subjects^18^. The need for recipient individual consent was waived by the institutional review board that approved this study with protocol number PRO 13060220.

## Results

### Patient characteristics of the study population before propensity-score matching

**Fig. 1:**
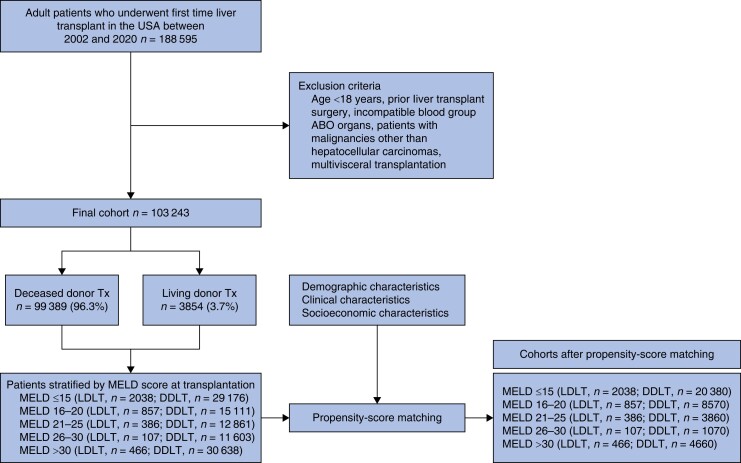
Flow chart of the study population. MELD, model for end-stage liver disease; LDLT, live donor liver transplantation; DDLT, deceased donor liver transplantation.

Among 188 595 patients who were screened for this study, 103 243 satisfied the inclusion criteria. The flowchart illustrating how the cohort was selected is reported in *Fig. 1*. DDLT recipients represented 96.3% of the study population (*n* = 99 389) whereas the remaining 3854 patients were LDLT recipients (3.7%). The baseline demographic and clinical characteristics of the study population before propensity-score matching are reported in *Table S1*. The median age at transplantation was 56 years (i.q.r. 49–62), most patients were male (65.8%) and the median MELD at transplantation was 20 (i.q.r. 14–28). The most common indication for DDLT was viral hepatitis (B or C; 22.6%), alcoholic cirrhosis (22.5%) and hepatocellular carcinoma (17.3%). The most common indication for LDLT was primary biliary or primary sclerosing cholangitis (26.9%), viral hepatitis (B or C; 17.4%) and metabolic fatty liver disease (13.9%). The median MELD at the time of LT was 14 for LDLT recipients (i.q.r. 11–18) and 20 for DDLT recipients (i.q.r. 14–29) (*P* < 0.001). LDLT recipients had a higher performance status (Karnofsky 70% *versus* 60%; *P* < 0.001), a higher level of education (college–university degree 54.9% *versus* 45.3%) and were less likely from ethnic-racial minorities (8.1% *versus* 15%; *P* < 0.001) and more likely holders of private health insurance (68.8% *versus* 55.19%; *P* < 0.001). History of diabetes, portal vein thrombosis, need for life support and dialysis were also less common in LDLT recipients. Live donors were younger than deceased donors (median 37 years *versus* 41 years, *P* < 0.001) and the median difference in CIT between LDLT and DDLT recipients was 4.5 h (median CIT = 1.6 h for LDLT *versus* 6.1 h for DDLT, *P* < 0.001). Side-to-side comparisons of the characteristics of the two groups stratified by MELD scores are reported in *Table S2a–e*.

### Patient survival before propensity-score matching stratified by model for end-stage liver disease

The median follow-up for the entire population was 13.3 years (95% c.i. 13.2 to 13.5). The median follow-up for LDLT recipients was 13.2 years (95% c.i. 13.0 to 13.4) whereas the median follow-up for DDLT was 16.9 years (95% c.i. 15.7 to 18.1). *Table S3* summarizes the causes of death after LDLT *versus* DDLT.

Unadjusted survival analysis showed that patients who received a LDLT had a significantly higher 5-year patient survival rate (81.4%; 95% c.i. 81.2 to 81.6) in comparison to DDLT recipients (75.5%; 95% c.i. 75.1 to 75.7) (*P* < 0.001; *Fig. S1a*). After stratification by MELD score, the patient survival advantage of LDLT recipients was statistically significant only for patients transplanted with MELD ≤25 (all *P* ≤ 0.001; *Fig. S1b–d*). No statistically significant differences in patient survival were observed between the two groups when the MELD was 26–30 (*P* = 0.139; *Fig. S1e*). On the other hand, when MELD was > 30, LDLT was associated with a significantly lower 5-year patient survival (60.0% (95% c.i. 59.5 to 60.5) in comparison to DDLT (73.9% (95% c.i. 73.4 to 74.4); *P* < 0.024; *Fig. S1f*). After adjusting for differences in recipients’ and donors’ characteristics in a multivariate Cox-regression analysis, the type of LT (LDLT *versus* DDLT) did not emerge as an independent predictor for patient survival (aHR = 1.07; 95% c.i. 0.95 to 1.20; *P* = 0.263; *Table S4*).

### Characteristics of the study population after propensity score matching

Propensity-score matching paired 3854 LDLT patients with 31 522 DDLT recipients with similar characteristics (*Table 1*). The list of parameters that were used to pair LDLT with DDLT patients and the standardized mean differences between the two groups measured before and after propensity-score matching are summarized in *Table S5a–e*. Although significantly reduced, propensity-score matching was unable to completely alleviate some of the differences between LDLT and DDLT recipients, particularly for patients belonging to the low MELD strata (MELD ≤ 15). As reported in *Table S5a–e*, the number of parameters that were still statistically significantly different between LDLT and DDLT patients after propensity-score matching were 11, 7 and 1 for patients with MELD ≤15, 16–20 and 21–26 respectively.

**Table 1:** Characteristics of the cohort of adult recipients of first-time liver transplantation in the USA between 1 January 2002 and 31 December 2020, after propensity-score matching stratified by the model for end-stage liver disease at the time of surgery

Variable	DDLT	LDLT	*P*
	*n* = 31 522	*n* = 3854	
**Recipients**			
Female	10 933 (34.6)	1749 (45.4)	<0.001
Age (years), median (i.q.r.)	54.8 (49–62)	53.3 (46–63)	<0.001
**Primary indication for transplant, *n* (%)**			
Acute liver failure	1260 (4.0)	56 (1.5)	<0.001
Alcohol-induced cirrhosis	7092 (22.5)	513 (13.3)	
Metabolic fatty liver disease	3404 (10.8)	537 (13.9)	
Primary biliary cirrhosis/primary sclerosing cholangitis	2805 (8.9)	1035 (26.9)	
Hepatocellular carcinoma	5453 (17.3)	471 (12.2)	
Viral hepatitis	7124 (22.6)	671 (17.4)	
Other	4381 (13.9)	571 (14.8)	
**MELD, median (i.q.r.)**	20.6 (14–29)	14 (11–18)	<0.001
MELD ≤15	15 537 (49.2)	2038 (52.9)	<0.001
MELD 16–20	8162 (25.8)	857 (22.2)	
MELD 21–25	3708 (11.8)	386 (10.0)	
MELD 26–30	875 (2.8)	107 (2.8)	
MELD>30	3240 (10.3)	466 (1.5)	
**Race/ethnicity, *n* (%)**			<0.001
White/Caucasian	25 533 (81.0)	3156 (82.1)	<0.001
Black/African American	978 (3.1)	138 (3.6)	
Hispanic/Latinos	3960 (12.5)	421 (10.9)	
Asian	1040 (3.2)	102 (2.6)	
Other/unknown	41 (0.1)	37 (1.0)	
**Karnofsky perfomance status, median (i.q.r.)**	66.8 (30–80)	70.0 (50–80)	<0.001
**Highest education level, *n* (%)**			
Elementary or unknown	3909 (12.4)	438 (11.4)	<0.001
Grade school	977 (3.1)	106 (2.8)	
High school	9866 (31.3)	1195 (31.0)	
College or university	16 770 (53.2)	2115 (54.9)	
**Primary health insurance, *n* (%)**			
Private	21 782 (69.1)	2692 (69.8)	<0.001
Medicare/Medicaid	8542 (27.1)	1062 (27.6)	
Public	851 (2.7)	44 (1.1)	
Other	347 (1.1)	56 (1.5)	
**Recipient blood group, *n* (%)**			
O	14 216 (45.1)	1774 (46.0)	<0.001
A	13 176 (41.8)	1615 (41.9)	
B	3499 (11.1)	400 (10.4)	
AB	630 (2.0)	65 (1.7)	
**BMI, median (i.q.r.)**	26.1 (23.4–31.1)	26 (23.3–30)	<0.001
<18.5	662 (2.1)	77 (2.0)	<0.001
18.5–24.9	11 096 (35.2)	1429 (37.1)	
25–29.9	11 884 (37.7)	1388 (36.0)	
≥30	7881 (25.0)	960 (24.9)	
History of dialysis, *n* (%)	2522 (0.8)	22 (0.6)	<0.001
History of diabetes, *n* (%)	7439 (23.6)	837 (21.7)	<0.001
History of TIPSS, *n* (%)	2932 (9.3)	303 (7.9)	0.008
History of portal vein thrombosis, *n* (%)	2742 (8.7)	364 (9.4)	0.003
Need for life support before transplantation, *n* (%)	662 (2.1)	36 (0.9)	<0.001
Patient on ventilator before transplantation, *n* (%)	473 (1.5)	19 (0.5)	<0.001
History of spontaneous bacterial peritonitis, *n* (%)	1072 (3.4)	107 (2.8)	<0.001
Prior abdominal surgery, *n* (%)	18 188 (57.7)	2143 (55.6)	0.003
**Donors**			
Female, *n* (%)	15 824 (50.2)	2022 (52.5)	<0.001
Age (years), median (i.q.r.)	39.2 (27–54)	37 (29–46)	<0.001
BMI, median (i.q.r.)	27.3 (23.1–30.7)	26 (23.6–28.8)	<0.001
**Cold ischaemia time (hours), median (i.q.r.)**	4.3 (2.9–6)	2 (1.0–2.2)	<0.001
0–6 h, *n* (%)	48 327 (88.2)	3800 (98.6)	<0.001
6.1–12 h, *n* (%)	48 349 (11.0)	32 (0.8)	
>12 h, *n* (%)	2713 (1.0)	22 (0.6)	

Values are *n* (%) unless otherwise stated. TIPSS, trans-hepatic portosystemic shunt; MELD, model for end-stage liver disease; LDLT, live donor liver transplantation; DDLT, deceased donor liver transplantation; i.q.r., interquartile range.

### Patient survival after propensity-score matching

**Fig. 2:**
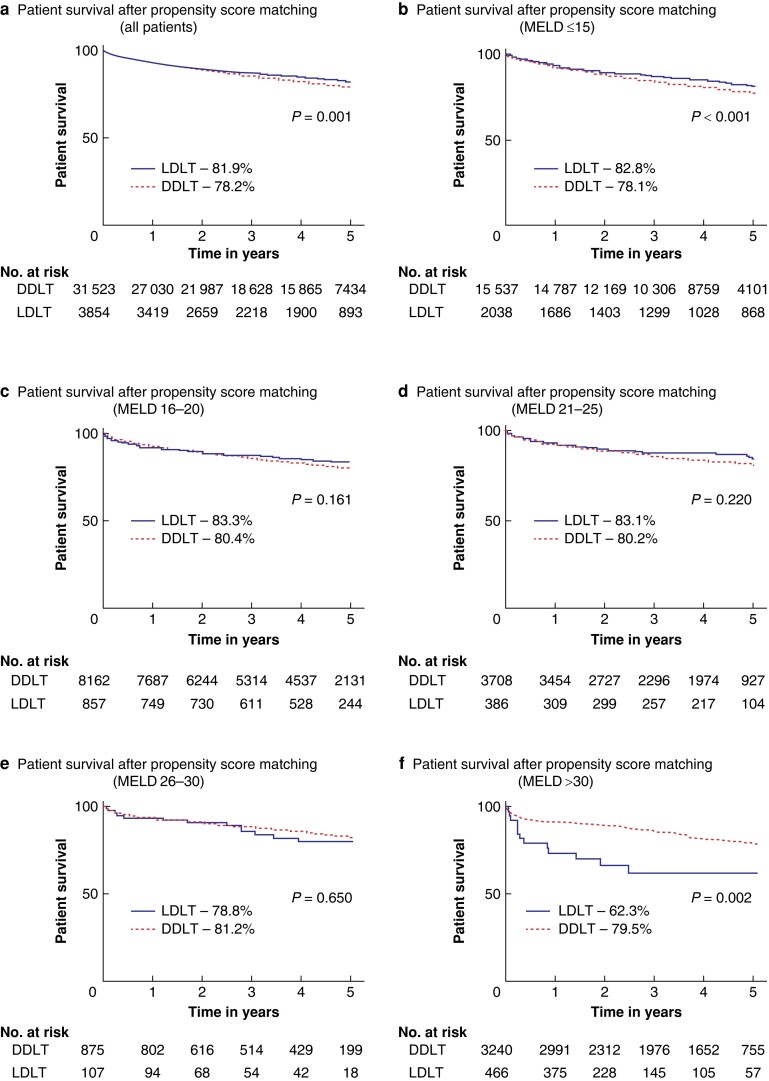
Kaplan–Meier functions obtained after propensity-score matching representing patient survival after live donor liver transplantation (LDLT) *versus* deceased donor liver transplantation (DDLT) stratified by the model for end-stage liver disease (MELD) at the time of surgery **a** includes the cohort of all patients who were matched using propensity score irrespective of their MELD score at the time of surgery. **b**–**f** represent the survival functions of the two groups after propensity-score matching stratified by MELD score obtained the day of surgery.

**Fig. 3:**
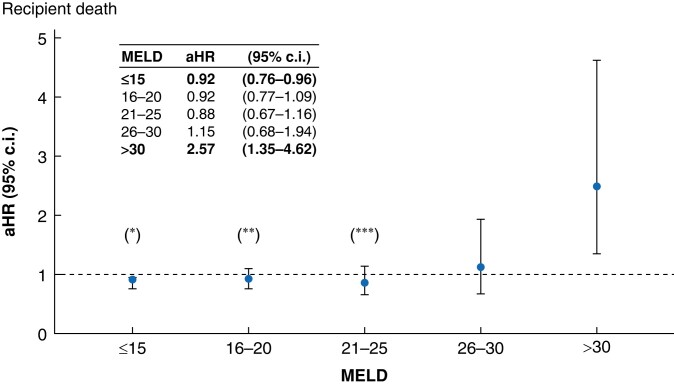
Fully adjusted hazard ratios (aHR) for death after propensity-score matching of patients who underwent live donor liver transplantation (LDLT) *versus* deceased donor liver transplantation (DDLT) stratified by model for end-stage liver disease (MELD) score at the time of surgery Recipients of DDLT represented the reference group. Values in bold represent statistically significant differences between the two groups (*P* < 0.05). *HR adjusted for recipient sex, recipient age, recipient race/ethnicity, recipient BMI, recipient highest degree of education, type of health insurance, indication for liver transplantation, donor sex, donor BMI, donor age, year of transplantation. **HR adjusted for recipient sex, recipient age, recipient BMI, donor sex, donor BMI, donor age, year of transplantation. ***HR adjusted for recipient functional status.

Survival analysis after propensity-score matching revealed that LDLT recipients had a superior 5-year survival rate than DDLT recipients (81.9% *versus* 78.2%; *P* < 0.001; *Fig. 2a*). The survival benefit of LDLT was also statistically significant for patients with MELD ≤15 (82.8% *versus* 78.1%; *P* < 0.001; *Fig. 2b*). For patients with MELD 16–30, there were no significant survival differences between the two groups (*Fig. 2c–e*). On the other hand, for individuals with MELD > 30, LDLT was associated with a significantly lower 5-year patient survival (62.3% *versus* 79.5%; *P* = 0.002; *Fig. 2f*). To account for the diminished but persistent differences in characteristics between patients belonging to the LDLT and DDLT groups for each MELD stratum, Cox-regression models were used to adjust for those imbalances. The risk of death after LDLT was significantly lower than DDLT for patients with MELD ≤15 (aHR = 0.92; 95% c.i. 0.76 to 0.96; *P* = 0.013). For patients with MELD scores ranging from 16 to 30, there was equipoise between the two transplant modalities. Conversely, patients with MELD scores exceeding 30 exhibited a significantly higher risk of death following LDLT (HR 2.57; 95% c.i. 1.35 to 4.62; *P* = 0.003; *Fig. 3*).

### Death-censored graft failure after propensity-score matching

**Fig. 4:**
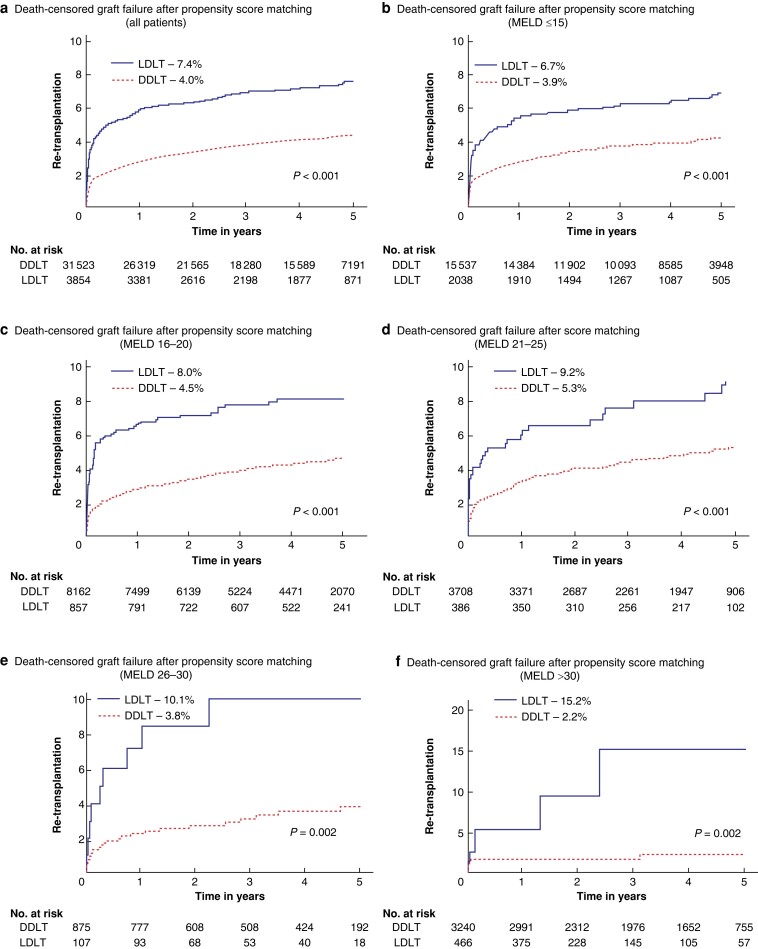
Kaplan–Meier functions of death-censored graft failure obtained after propensity-score matching of recipients of live donor liver transplantation (LDLT) *versus* deceased donor liver transplantation (DDLT) stratified by model for end-stage liver disease (MELD) at the time of surgery **a** includes all patients who were matched by propensity score irrespective of their MELD at the time of surgery. **b**–**f** represent the death-censored graft failure functions of the two groups after propensity score matching stratified by MELD scores at the time of surgery.

**Fig. 5:**
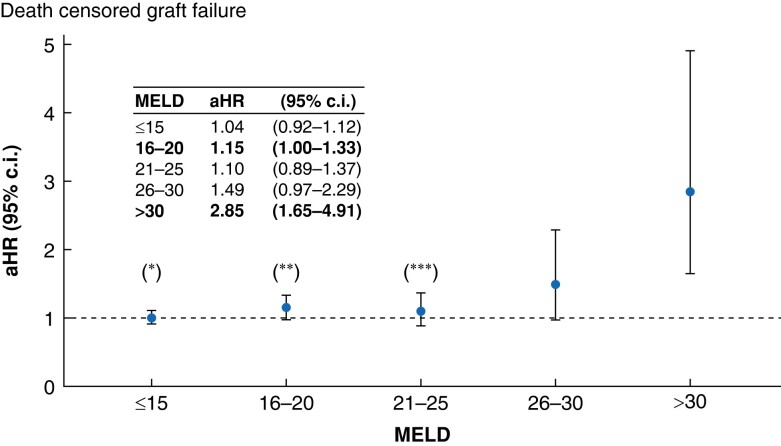
Fully adjusted hazard ratios (aHR) for death-censored graft failure after propensity-score matching of live donor liver transplant (LDLT) and deceased donor liver transplant (DDLT) recipients, stratified by model for end-stage liver disease (MELD) score at the time of surgery DDLT recipients represented the reference group for each MELD stratum. Statistically significant differences are reported in bold. *HR adjusted for recipient sex, recipient age, recipient race/ethnicity, recipient BMI, recipient highest degree of education, type of health insurance, indication for liver transplantation, donor sex, donor BMI, donor age, year of transplantation. **HR adjusted for recipient sex, recipient age, recipient BMI, donor sex, donor BMI, donor age, year of transplantation. ***HR adjusted for recipient functional status.

After propensity-score matching, the overall restricted 5-year death-censored graft failure rate was 4.3% with 285 (7.4%) failures observed after LDLT and 1256 (4.0%) after DDLT (*P* < 0.001; *Fig. 4a*). Among patients with failed grafts, 948 (65.4%) underwent re-transplantation within the first year, including 208 (5.4%) LDLT recipients and 788 (2.5%) DDLT recipients (*P* < 0.001). The unadjusted death-censored graft failure rate was consistently higher among LDLT recipients irrespective of their MELD score at the time of their index operations (all *P* < 0.05; *Fig. 4b–f*). After accounting for parameters that were still statistically significantly different after propensity-score matching, no statistically significant differences between the two groups were observed for patients with MELD≤15 and for patients with MELD = 21–30. For patients with MELD scores of 16–20 and those with MELD scores exceeding 30, however, LDLT was associated with a statistically significant higher risk of death-censored graft failure in comparison to DDLT (aHR = 1.15; 95% c.i. 1.00 to 1.33; *P* = 0.04 and HR = 2.85; 95% c.i. 1.65–4.91; *P* = 0.001 respectively; *Fig. 5*).

### Sensitivity analysis

**Fig. 6:**
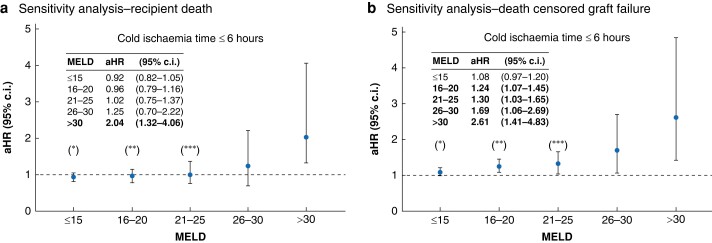
**a** Fully adjusted hazard ratios (aHR) for death after propensity score matching of live donor liver transplant (LDLT) and deceased donor liver transplantation (DDLT) recipients, stratified by model for end-stage liver disease (MELD), transplanted with cold ischaemia time ≤6 h. Recipients of DDLT represented the reference group. Statistically significant differences are reported in bold. **b** Fully adjusted hazard ratios (aHR) for death-censored graft failure after propensity-score matching of live donor liver transplant (LDLT) and deceased donor liver transplant (DDLT) recipients, stratified by model for end-stage liver disease (MELD), transplanted with cold ischaemia time ≤6 h. Recipients of DDLT represented the reference group. Statistically significant differences are reported in bold. *HR adjusted for recipient sex, recipient age, recipient race/ethnicity, recipient BMI, recipient highest degree of education, type of health insurance, indication for liver transplantation, donor sex, donor BMI, donor age, year of transplantation. **HR adjusted for recipient sex, recipient age, recipient BMI, donor sex, donor BMI, donor age, year of transplantation. ***HR adjusted for recipient functional status.

After propensity-score matching, if CIT was <6 h, no significant differences in patient survival were found between LDLT and DDLT for MELD scores <30 (*Fig. 6a*). Conversely, for patients with MELD scores ≥30, LDLT was associated with a significantly higher risk of death (aHR 2.04; 95% c.i. 1.32 to 4.06; *P* < 0.001). The risk of graft failure, on the other hand, was significantly higher after LDLT for patients with MELD scores as low as 15 if transplanted with CIT < 6 h (*Fig. 6b*).

## Discussion

In this study, we undertook a comparative analysis of postoperative outcomes between LDLT recipients and those who underwent DDLT. To account for the significant differences in sociodemographic and clinical characteristics between the two groups, our approach involved the use of propensity-score matching stratified by the MELD score at the time of surgery. The most relevant finding of our study is that for patients with MELD scores exceeding 30, outcomes are significantly worse after LDLT in comparison to DDLT. Equipoise between the two modalities was observed for patients with MELD scores between 16 and 30. On the other hand, for patients with MELD ≤ 15, LDLT was associated with superior survival rates. The results of our study were dependent on the duration of CIT, however. For patients transplanted with CIT ≤ 6 h, the survival advantage provided by LDLT, especially for recipients with low MELD scores, was no longer significant. This observation suggests that the better survival of LDLT recipients might be due to the significantly shorter anoxic stress of the grafts as LDLT is performed electively with fewer logistic challenges that often determine the duration of CIT. Conversely, the lower patient survival observed among LDLT recipients with MELD > 30 was not sensitive to changes in the duration of CIT, suggesting that for patients with high acuity of liver disease, the use of partial grafts might be inadequate.

To the best of our knowledge, our study is the first to investigate whether there is equipoise between LDLT and DDLT recipients across different MELD scores. Previous studies have compared the outcomes after LDLT and DDLT^3,7^ and, through propensity-score matching, it was found that there were no significant differences in patient survival between the two groups^7^. However, these studies did not conclusively demonstrate survival equipoise between the two modalities for patients with increasing MELD scores. In a recent study from the United States, Jackson *et al*.^3^ found that after one year from the date of listing, LDLT provided a survival benefit to patients with MELD-Na as low as 11. In another study from Samsung Medical Center in South Korea^19^, Oh and colleagues found that after adjusting for several confounders, LDLT provided a survival advantage in comparison to best supportive therapy in patients with MELD-Na as low as 13. In both studies^3,19^, survival was measured from the time of listing rather than from the time of surgery, and the investigators did not use propensity-score matching to pair patients with similar characteristics. In addition, it is important to notice that in the United States, many LDLT recipients are listed immediately before their surgery. This trend, therefore, biases the results in favor of LDLT. Similar to a study by Cotter *et al*.^7^, we observed a higher rate of graft failure and re-transplantation rates after LDLT. They^7^ reported that, within the first year after LT, re-transplantation rate was 5.3% in LDLT recipients *versus* 2.3% in DDLT recipients (*P* < 0.001). In our study, we confirmed that re-transplantation rates at 5 years after LT were 7.4% after LDLT and 4.0% after DDLT (*P* < 0.001). We also noted that the risk of death-censored graft failure was consistently higher after LDLT across all the MELD strata. Unfortunately, due to insufficient granular data on the primary cause of graft failure reported in the SRTR, we were unable to perform further analyses to better identify the main reasons for the higher risk of graft loss observed after LDLT. Based on previous studies, however, we speculate that among the most plausible reasons for the higher rate of graft failure among LDLT recipients was their higher susceptibility to experience arterial or venous thrombosis due to the smaller calibre of the anastomoses. Moreover, these recipients face a greater likelihood of biliary complications, which can further compromise graft viability. Another possible cause of graft failure after LDLT is ‘small for size syndrome’ observed when an insufficient functional liver mass is transplanted. The limitations of the current study, however, underscore the need for further investigations on this important aspect of LT, as it would be critical to better elucidate the main factors that contribute to the higher risk of graft failure after LDLT and the higher risk of death for patients with high MELD scores. Such studies could enhance our understanding of recipient or donor factors that might influence graft and patient survival after LDLT and inform strategies to mitigate the risks of poor outcomes.

Despite the above limitations, our study stands as one of the largest to analyse the outcomes of LDLT recipients in North America using propensity-score matching. As our primary aim was to test whether postoperative outcomes of LDLT *versus* DDLT recipients were equivalent in patients with different acuity of liver disease, a large sample size was needed. Therefore, to assess equipoise between LDLT and DDLT for patients belonging to different MELD groups, we used data on patients transplanted between 2002 and 2020, recognizing that over this period advances in the care and selection of LT recipients and their donors have inevitably occurred. One of the strengths of our study is that its findings are generalizable to transplant programmes across the country as we used data extracted from a national registry that includes all recipients of LT in the United States. However, it is important to note that only a select few transplant centres perform LDLTs and there is a considerable variation in the number of LDLTs performed at each centre and expertise in this operation. Additionally, the retrospective design of our study introduced the risk of selection bias that could not be completely removed even with the use of propensity-score matching. Lastly, the relatively small number of patients with high MELD scores who underwent LDLT raises concerns that our findings are far less robust for this subgroup of patients. Therefore, future studies with a larger number of high MELD score patients treated with LDLT will be necessary to confirm our findings. Also, in more recent years, the MELD-Na has been introduced to prioritize patients in need of LT in the United States. As the MELD-Na has replaced the MELD score, it would be important to assess whether the results of this study hold true using the MELD-Na rather than the MELD score for the stratification of patients undergoing liver transplantation.

In conclusion, the findings of this study underscore the importance of using caution when recommending LDLT to patients with high MELD scores as patient and graft survival were significantly lower than the outcomes of DDLT recipients. Further studies are needed to validate our findings using data from patients transplanted in other countries, and efforts should be carried out to better understand the main reasons for the higher risk of graft loss observed after LDLT.

## Supplementary Material

zrae058_Supplementary_Data

## Data Availability

Data will be made available upon request.

## References

[1] Roll GR, Parekh JR, Parker WF, Siegler M, Pomfret EA, Ascher NL et al Left hepatectomy *versus* right hepatectomy for living donor liver transplantation: shifting the risk from the donor to the recipient. Liver Transpl 2013;19:472–481 23447523 10.1002/lt.23608

[2] Wong TCL, Fung JYY, Pang HH, Leung CKL, Li HF, Sin SL et al Analysis of survival benefits of living *versus* deceased donor liver transplant in high model for end-stage liver disease and hepatorenal syndrome. Hepatology 2021;73:2441–2454 33006772 10.1002/hep.31584 PMC8252626

[3] Jackson WE, Malamon JS, Kaplan B, Saben JL, Schold JD, Pomposelli JJ et al Survival benefit of living-donor liver transplant. JAMA Surg 2022;157:926 35921119 10.1001/jamasurg.2022.3327 PMC9350845

[4] Humar A, Ganesh S, Jorgensen D, Tevar A, Ganoza A, Molinari M et al Adult living donor *versus* deceased donor liver transplant (LDLT *versus* DDLT) at a single center: time to change our paradigm for liver transplant. Ann Surg 2019;270:444–451 31305283 10.1097/SLA.0000000000003463

[5] Dew MA, Butt Z, Humar A, DiMartini AF. Long-term medical and psychosocial outcomes in living liver donors. Am J Transplant 2017;17:880–892 27862972 10.1111/ajt.14111 PMC5510163

[6] Berg CL, Merion RM, Shearon TH, Olthoff KM, Brown RS Jr, Baker TB et al Liver transplant recipient survival benefit with living donation in the model for endstage liver disease allocation era. Hepatology 2011;54:1313–1321 21688284 10.1002/hep.24494 PMC3184197

[7] Cotter TG, Minhem M, Wang J, Peeraphatdit T, Ayoub F, Pillai A et al Living donor liver transplantation in the United States: evolution of frequency, outcomes, center volumes, and factors associated with outcomes. Liver Transpl 2021;27:1019–1031 33619854 10.1002/lt.26029 PMC9257956

[8] Kwong AJ, Kim WR, Lake JR, Smith JM, Schladt DP, Skeans MA et al OPTN/SRTR 2019 annual data report: liver. Am J Transplant 2021;21:208–315 10.1111/ajt.16494 33595192

[9] Kamath PS, Wiesner RH, Malinchoc M, Kremers W, Therneau TM, Kosberg CL et al A model to predict survival in patients with end-stage liver disease. Hepatology 2001;33:464–470 11172350 10.1053/jhep.2001.22172

[10] Testa G, Malago M, Nadalin S, Hertl M, Lang H, Frilling A et al Right-liver living donor transplantation for decompensated end-stage liver disease. Liver Transpl 2002;8:340–346 11965577 10.1053/jlts.2002.32941

[11] Hart A, Schladt DP, Zeglin J, Pyke J, Kim WR, Lake JR et al Predicting outcomes on the liver transplant waiting list in the United States: accounting for large regional variation in organ availability and priority allocation points. Transplantation 2016;100:2153–2159 27490411 10.1097/TP.0000000000001384 PMC5369025

[12] Merion RM, Schaubel DE, Dykstra DM, Freeman RB, Port FK, Wolfe RA. The survival benefit of liver transplantation. Am J Transplant 2005;5:307–313 15643990 10.1111/j.1600-6143.2004.00703.x

[13] World Health Organization . *Body Mass Index—BMI*. https://www.who.int/data/gho/data/themes/topics/topic-details/GHO/body-mass-index (accessed 3 December 2023)

[14] Caldwell BM, Graham FK, Greenman M, Hartmann AF, Pennoyer MM. Relationship between clinical status and behavior test performance in a newborn group with histories suggesting anoxia. J Pediatr 1957;50:177–189 13385775 10.1016/s0022-3476(57)80050-x

[15] Kaplan EL, Meier P. Nonparametric estimation from incomplete observations. J Am Stat Assoc 1958;53:457–481

[16] Hemmila MR, Birkmeyer NJ, Arbabi S, Osborne NH, Wahl WL, Dimick JB. Introduction to propensity scores: a case study on the comparative effectiveness of laparoscopic vs open appendectomy. Arch Surg 2010;145:939–945 20956761 10.1001/archsurg.2010.193

[17] von Elm E, Altman DG, Egger M, Pocock SJ, Gotzsche PC, Vandenbroucke JP et al The strengthening the reporting of observational studies in epidemiology (STROBE) statement: guidelines for reporting observational studies. Lancet 2007;370:1453–1457 18064739 10.1016/S0140-6736(07)61602-X

[18] The Helsinki Declaration of the World Medical Association. Gac Med Mex 2001;137:387–390 11519365

[19] Oh N, Kim JM, Han S, Jo SJ, An S, Park S et al Survival after living donor liver transplantation versus best supportive care in patients with end-stage liver disease with various MELD-Na scores: retrospective cohort study. BJS Open 2023;7:zrad127 38011798 10.1093/bjsopen/zrad127 PMC10681711

