# Supplementary material for: Survival after live donor versus deceased donor liver transplantation: propensity score–matched study
Source: BJS Open. 2024 Jun 5;8(3):zrae058. doi: 10.1093/bjsopen/zrae058 (PMC11152206; doi:10.1093/bjsopen/zrae058)
Supplement: zrae058_Supplementary_Data [file zrae058_supplementary_data.docx]

**Survival after Live Donor Versus Deceased Donor Liver Transplantation: A Propensity Score Matched Analysis**

Authors:

Christof Kaltenmeier^1^ MD^1^, Hao Liu MD, PhD^1^, Zhang, Xingyu PhD^2^, Armando Ganoza^1^ MD, MBA, Andrew Crane^1^ MD, Colin Powers^1^ MD, Vikraman Gunabushanam^1^ MD, Jaideep Behari^3^, MD, PhD, Michele Molinari^1^ MD, MHS^1^

**Affiliations:**

1. Department of Surgery, University of Pittsburgh Medical Center, Pittsburgh, Pennsylvania, United States
2. Department of Biostatistics, University of Pittsburgh Medical Center, Pittsburgh, Pennsylvania, United States

3 Department of Medicine, University of Pittsburgh Medical Center, Pittsburgh, Pennsylvania, United States

**Corresponding author.**

Name: Michele Molinari, MD, MHS,

Professor of Surgery, Division of Abdominal Transplant Surgery,

Address: University of Pittsburgh Medical Center, Montefiore Hospital, 3459 Fifth Avenue, N758, Pittsburgh, Pennsylvania, 15213, United States.

Email address: [molinarim@upmc.edu](mailto:molinarim@upmc.edu),

Fax 412-864-5458

**ORCID ID**: 0000-0002-9763-6253

**Twitter: N.A.**

**Table of contents:**

**Supplementary Table 1 – Pages 2-3**

**Supplementary Table 2 – Pages 4-13**

**Supplementary Table 3 – Page 14**

**Supplementary Table 4 – Pages 15-17**

**Supplementary Table 5 – Pages 18-22**

**Supplementary Figure 1 – Page 23**

| **Supplementary Table 1** Sociodemographic and clinical characteristics of the cohort of adult patients who underwent first-time liver transplantation in the United States between January 1, 2002, and December 31, 2020, before propensity score matching.   \| **Variable** \| **Entire Cohort** \| \| **DDLT** \| \| **LDLT** \| \| ***P Value*** \| \| --- \| --- \| --- \| --- \| --- \| --- \| --- \| --- \| \| n=103,243 \| \| n=99,389 \| \| n=3,854 \| \| \| **Recipients** \|  \|  \|  \|  \|  \|  \|  \| \| Female, n. (%) \| 35,286 \| 34.2% \| 33,537 \| 33.7% \| 1749 \| 45.4% \| <0.001 \| \| Age, years, median (IQR) \| 56.0 \| 49-62 \| 56 \| 49-62 \| 56 \| 46-63 \| <0.001 \| \| **Primary Indication for transplant, n. (%)** \|  \|  \|  \|  \|  \|  \|  \| \| Acute liver failure \| 4,076 \| 3.9% \| 4,020 \| 4.0% \| 56 \| 1.5% \| <0.001 \| \| Alcohol-induced cirrhosis \| 22,888 \| 22.2% \| 22,375 \| 22.5% \| 513 \| 13.3% \| \| Nonalcoholic fatty liver disease \| 11,309 \| 11.0% \| 10,772 \| 10.8% \| 537 \| 13.9% \| \| Primary biliary cirrhosis / Primary sclerosing cholangitis \| 9,837 \| 9.5% \| 8,802 \| 8.9% \| 1,035 \| 26.9% \| \| Hepatocellular carcinoma \| 17,682 \| 17.1% \| 17,211 \| 17.3% \| 471 \| 12.2% \| \| Viral hepatitis \| 23,099 \| 22.4% \| 22,428 \| 22.6% \| 671 \| 17.4% \| \| Other \| 14,352 \| 13.9% \| 13,781 \| 13.9% \| 571 \| 14.8% \|  \| \| **MELD median (IQR)** \| 20.0 \| 14-28 \| 20.0 \| 14-29 \| 14 \| 11-18 \| <0.001 \| \| MELD ≤ 15 \| 31,214 \| 30.2% \| 29,176 \| 29.4% \| 2,038 \| 52.9% \| <0.001 \| \| MELD 16-20 \| 15,968 \| 15.5% \| 15,111 \| 15.2% \| 857 \| 22.2% \| \| MELD 21-25 \| 13,247 \| 12.8% \| 12,861 \| 12.9% \| 386 \| 10.0% \| \| MELD 26-30 \| 11,710 \| 11.3% \| 11,603 \| 11.7% \| 107 \| 2.8% \| \| MELD >30 \| 31,104 \| 30.1% \| 30,638 \| 30.8% \| 466 \| 1.5% \| \| **Performance Status, Karnofsky Score, median (IQR)** \| 60 \| 30-70 \| 60 \| 30-70 \| 70 \| 50-80 \| <0.001 \| \| **Race/Ethnicity, n. (%)** \|  \|  \|  \|  \|  \|  \| <0.001 \| \| White/Caucasian \| 88,020 \| 85.26% \| 84,443 \| 85.00% \| 3577 \| 92.80% \| <0.001 \| \| Black/African American \| 9,484 \| 9.19% \| 9,346 \| 9.40% \| 138 \| 3.60% \| \| Hispanic/Latinos \|  \|  \|  \|  \|  \|  \| \| Asian \| 4,332 \| 4.20% \| 4,230 \| 4.30% \| 102 \| 2.6% \| \| Other/unknown \| 1,407 \| 1.36% \| 1,370 \| 1.40% \| 37 \| 1.0% \| \| **Karnofsky Performance Status, median (IQR)** \| 60.0 \| 30-70 \| 60 \| 30-80 \| 70.0 \| 50-80 \| <0.001 \| \| **Highest education level, n. (%)** \|  \|  \|  \|  \|  \|  \|  \| \| Elementary or unknown \| 11,036 \| 10.69% \| 10,598 \| 10.7% \| 438 \| 11.4% \| <0.001 \| \| Grade school \| 4,541 \| 4.40% \| 4,435 \| 4.5% \| 106 \| 2.8% \| \| High school \| 40,533 \| 39.3% \| 39,338 \| 39.6% \| 1195 \| 31.0% \| \| College or University \| 47,133 \| 45.7% \| 45,018 \| 45.3% \| 2115 \| 54.9% \| \| **Primary health insurance, n. (%)** \|  \|  \|  \|  \|  \|  \|  \| \| Private \| 57,459 \| 55.7% \| 54,767 \| 55.1% \| 2692 \| 69.8% \| <0.001 \| \| Medicare/Medicaid \| 40,328 \| 39.1% \| 39,266 \| 39.5% \| 1062 \| 27.6% \| \| Public \| 3,382 \| 3.3% \| 3,338 \| 3.4% \| 44 \| 1.1% \| \| Other \| 2,074 \| 2.0% \| 2,018 \| 2.0% \| 56 \| 1.5% \| \| **Recipient Blood group, n. (%)** \|  \|  \|  \|  \|  \|  \|  \| \| O \| 46,126 \| 44.7% \| 44,352 \| 44.6% \| 1774 \| 46.0% \| <0.001 \| \| A \| 38,111 \| 36.9% \| 36,496 \| 36.7% \| 1615 \| 41.9% \| \| B \| 13,910 \| 13.5% \| 13,510 \| 13.6% \| 400 \| 10.4% \| \| AB \| 5,096 \| 4.9% \| 5,031 \| 5.1% \| 65 \| 1.7% \| \| **BMI, median, IQR** \| 27.7 \| 24.3-32 \| 27.8 \| 24.3-32.1 \| 26.3 \| 23.3-30 \| <0.001 \| \| <18.5 \| 2,011 \| 1.9% \| 1,934 \| 1.9% \| 77 \| 2.0% \| <0.001 \| \| 18.5-24.9 \| 28,990 \| 28.1% \| 27,561 \| 27.7% \| 1,429 \| 37.1% \| \| 25-29.9 \| 35,721 \| 34.6% \| 34,333 \| 34.5% \| 1,388 \| 36.0% \| \| ≥30 \| 36,521 \| 35.4% \| 35,561 \| 35.8% \| 960 \| 24.9% \| \| History of dialysis, n. (%) \| 13,730 \| 13.3% \| 13,708 \| 13.8% \| 22 \| 0.6% \| <0.001 \| \| History of diabetes, n. (%) \| 27,328 \| 26.5% \| 26,491 \| 26.7% \| 837 \| 21.7% \| <0.001 \| \| History of TIPSS, n. (%) \| 9,369 \| 9.1% \| 9,066 \| 9.1% \| 303 \| 7.9% \| 0.008 \| \| History of Portal vein thrombosis, n. (%) \| 11,257 \| 10.9% \| 10,893 \| 11.0% \| 364 \| 9.4% \| 0.003 \| \| Need for life support before transplantation, n. (%) \| 8,578 \| 8.3% \| 8,542 \| 8.6% \| 36 \| 0.9% \| <0.001 \| \| Patient on mechanical ventilation before transplantation, n. (%) \| 5,479 \| 5.3% \| 5,460 \| 5.5% \| 19 \| 0.5% \| <0.001 \| \| History of spontaneous bacterial peritonitis, n. (%) \| 4,871 \| 4.7% \| 4,764 \| 4.8% \| 107 \| 2.8% \| <0.001 \| \| Prior abdominal surgery, n. (%) \| 59,835 \| 58.0% \| 57,692 \| 58.0% \| 2143 \| 55.6% \| 0.003 \| \| **Donors** \|  \|  \|  \|  \|  \|  \|  \| \| Female \| 41,929 \| 40.61% \| 39,907 \| 40.2% \| 2022 \| 52.5% \| <0.001 \| \| Age, median, IQR \| 41.0 \| 27-54 \| 41 \| 27-54 \| 37 \| 29-46 \| <0.001 \| \| BMI, median, IQR \| 26.4 \| 23.1-30.5 \| 27.4 \| 23.1-30.7 \| 26.3 \| 23.6-28.8 \| <0.001 \| \| **Cold Ischemia time, hours, median (IQR)** \| 6.0 \| 7.9-4.7 \| 6.1 \| 4.9-8 \| 1.58 \| 1.0-2.2 \| <0.001 \| \| 0-6 \| 52,127 \| 50.5% \| 48,327 \| 48.6% \| 3800 \| 98.6% \| <0.001 \| \| 6.1-12 \| 48,381 \| 46.9% \| 48,349 \| 48.6% \| 32 \| 0.8% \| \| >12h \| 2,735 \| 2.6% \| 2,713 \| 2.7% \| 22 \| 0.6% \|   **Legend**: LDLT=live donor liver transplant, DDLT=deceased donor liver transplant, MELD=model for end stage liver disease, BMI=body mass index   \| **Supplementary Table 2A:** Recipient and donor characteristics of patients transplanted with MELD score <15 before propensity score matching. \| \| \| \| \| \| \| --- \| --- \| --- \| --- \| --- \| --- \| \| **Characteristics** \| **DDLT** \| \| **LDLT** \| \| **P Value** \| \| ***N. of patients*** \| 29,176 \| \| 2,038 \| \| \| Female sex, n. (%) \| 8,545 \| 29.4% \| 960 \| 47.1% \| <0.001 \| \| Age, years, median (IQR) \| 58.0 \| 52-63 \| 57.0 \| 48-63 \| <0.001 \| \| **Etiology of liver disease, n. (%)** \|  \|  \|  \|  \| <0.001 \| \| Alcohol \| 3,480 \| 15.6 \| 209 \| 10.3% \| \| Nonalcoholic fatty liver disease \| 2,004 \| 6.9% \| 254 \| 12.5% \| \| Primary biliary cirrhosis / Primary sclerosing cholangitis \| 1,754 \| 6.0% \| 544 \| 26.7% \| \| Hepatocellular carcinoma \| 11,446 \| 39.2% \| 352 \| 17.3% \| \| Viral \| 7,222 \| 32.2% \| 360 \| 17.7% \| \| Other \| 3,527 \| 12.0% \| 319 \| 15.6% \| \| **Race, n. (%)** \|  \|  \|  \|  \| <0.001 \| \| White or Hispanic \| 24,543 \| 84.1% \| 1,895 \| 93.0% \| \| Black \| 2,319 \| 7.9% \| 61 \| 3.0% \| \| Asian \| 1,981 \| 6.8% \| 61 \| 3.0% \| \| Other/unknown \| 333 \| 1.1% \| 21 \| 1.0% \| \| **Highest level of Education, n. (%)** \|  \|  \|  \|  \| <0.001 \| \| Unknown \| 2831 \| 9.7% \| 225 \| 11.0% \| \| Grade 1-8 \| 1,415 \| 4.8% \| 52 \| 2.6% \| \| High school Degree \| 11,627 \| 29.6% \| 615 \| 30.2% \| \| College-University Degree \| 13,303 \| 45.6% \| 1,146 \| 56.2% \| \| **Primary Payer, n. (%)** \|  \|  \|  \|  \| <0.001 \| \| Private health insurance \| 17,104 \| 54.9% \| 1,528 \| 70.7% \| \| Medicare/Medicaid \| 12,343 \| 39.6% \| 578 \| 26.7% \| \| Public health insurance \| 1,051 \| 3.4% \| 23 \| 1.1% \| \| Other payers \| 676 \| 2.2% \| 33 \| 1.5% \| \| **Recipient blood group, n. (%)** \|  \|  \|  \|  \| <0.001 \| \| O \| 12,609 \| 43.2% \| 923 \| 45.3% \| \| A \| 10,583 \| 36.2% \| 846 \| 41.5% \| \| B \| 4,228 \| 14.5% \| 232 \| 11.4% \| \| AB \| 1,756 \| 6.0% \| 37 \| 1.8% \| \| BMI, median, IQR \| 28 \| 24.6-31.9 \| 26.4 \| 23.5-29.9 \| <0.001 \| \| Dialysis, n. (%) \| 155 \| 0.5% \| 3 \| 0.1% \| 0.361 \| \| Diabetes, n. (%) \| 9,302 \| 28.9% \| 520 \| 24.1 \| <0.001 \| \| TIPS, n. (%) \| 2,360 \| 7.6% \| 164 \| 8.0% \| 0.528 \| \| Portal vein thrombosis, n. (%) \| 3,172 \| 10.2% \| 196 \| 9.6% \| 0.032 \| \| Life support, n. (%) \| 295 \| 0.9% \| 12 \| 0.6% \| 0.034 \| \| Ventilator, n. (%) \| 158 \| 0.5% \| 4 \| 0.2% \| 0.018 \| \| Spontaneous Bacterial Peritonitis, n. (%) \| 582 \| 2.0% \| 4 \| 2.1% \| 0.586 \| \| Prior abdominal surgery, n. (%) \| 17,793 \| 57.1% \| 1,148 \| 53.1% \| 0.177 \| \| **Donor Characteristics** \|  \|  \|  \|  \|  \| \| Female Gender, n. (%) \| 12,649 \| 40.6% \| 1,112 \| 51.4% \| <0.001 \| \| Age, years, median (IQR) \| 44 \| 28-56 \| 37 \| 29-46 \| <0.001 \| \| BMI, median, (IQR) \| 26.6 \| 23.2-31.0 \| 26.1 \| 23.6-28.9 \| <0.001 \| \| Cold Ischemia time, hours, median, (IQR) \| 6.21 \| 4.9-8.0 \| 1.6 \| 1.0-2.3 \| <0.001 \|   **Legend**: LDLT=live donor liver transplant, DDLT=deceased donor liver transplant, MELD=model for end stage liver disease, BMI=body mass index   \| **Supplementary Table 2B:** Recipient and donor characteristics of patients transplanted with MELD score 16-20 before propensity score matching. \| \| \| \| \| \| \| --- \| --- \| --- \| --- \| --- \| --- \| \| **Characteristics** \| **DDLT** \| \| **LDLT** \| \| **P Value** \| \| ***N. of patients*** \| 15,111 \| \| 857 \| \| \| Female sex, n. (%) \| 6,120 \| 32.6% \| 370 \| 43.2% \| <0.001 \| \| Age, years, median (IQR) \| 56.0 \| 50-62 \| 56.0 \| 47-62 \| <0.001 \| \| Etiology of liver disease, n. (%) \|  \|  \|  \|  \| <0.001 \| \| Alcohol \| 3,392 \| 22.4% \| 132 \| 15.4% \| \| Nonalcoholic fatty liver disease \| 1,986 \| 13.1% \| 122 \| 14.2% \| \| Primary biliary cirrhosis / Primary sclerosing cholangitis \| 1,597 \| 10.6% \| 239 \| 27.9% \| \| Hepatocellular carcinoma \| 2,062 \| 13.6% \| 70 \| 8.2% \| \| Viral \| 3,562 \| 23.6% \| 158 \| 18.4% \| \| Other \| 3,019 \| 19.9% \| 136 \| 15.8% \| \| **Race, n. (%)** \|  \|  \|  \|  \| <0.001 \| \| White or Hispanic \| 13,323 \| 88.2% \| 789 \| 92.1% \| \| Black \| 1,179 \| 7.8% \| 39 \| 4.6% \| \| Asian \| 408 \| 2.7% \| 19 \| 2.2% \| \| Other/unknown \| 201 \| 1.3% \| 10 \| 1.2% \| \| **Highest level of Education, n. (%)** \|  \|  \|  \|  \| <0.001 \| \| Unknown \| 1,602 \| 10.6% \| 88 \| 10.3% \| \| Grade 1-8 \| 591 \| 3.9% \| 30 \| 3.5% \| \| High school Degree \| 6,119 \| 40.5% \| 280 \| 32.7% \| \| College-University Degree \| 6,799 \| 45.0% \| 459 \| 53.6% \| \| **Primary Payer, n. (%)** \|  \|  \|  \|  \| <0.001 \| \| Private health insurance \| 10,417 \| 55.4% \| 712 \| 68.3% \| \| Medicare/Medicaid \| 7,362 \| 39.2% \| 297 \| 28.5% \| \| Public health insurance \| 615 \| 3.3% \| 18 \| 1.7% \| \| Other payers \| 404 \| 2.1% \| 16 \| 1.5% \| \| **Recipient blood group, n. (%)** \|  \|  \|  \|  \| <0.001 \| \| O \| 6,481 \| 42.9% \| 383 \| 44.7% \| \| A \| 5,501 \| 36.4% \| 381 \| 44.5% \| \| B \| 2,209 \| 14.6% \| 79 \| 9.2% \| \| AB \| 920 \| 6.1% \| 14 \| 1.6% \| \| BMI, median, IQR \| 27.8 \| 24.4-32.0 \| 26.2 \| 23.3-30.0 \| <0.001 \| \| Dialysis, n. (%) \| 853 \| 4.5% \| 2 \| 0.2% \| <0.001 \| \| Diabetes, n. (%) \| 5,233 \| 27.8% \| 207 \| 19.8% \| <0.001 \| \| TIPS, n. (%) \| 2,308 \| 12.3% \| 85 \| 8.1% \| <0.001 \| \| Portal vein thrombosis, n. (%) \| 1,949 \| 12.9% \| 115 \| 11.0% \| 0.108 \| \| Life support, n. (%) \| 378 \| 2.5% \| 12 \| 1.2% \| 0.006 \| \| Ventilator, n. (%) \| 232 \| 1.5% \| 7 \| 0.8% \| 0.087 \| \| Spontaneous Bacterial Peritonitis, n. (%) \| 674 \| 4.5% \| 36 \| 4.2% \| 0.516 \| \| Prior abdominal surgery, n. (%) \| 10,835 \| 57.6% \| 606 \| 58.1% \| 0.769 \| \| **Donor Characteristics** \|  \|  \|  \|  \|  \| \| Female Sex, n. (%) \| 7,547 \| 40.1% \| 560 \| 53.7% \| <0.001 \| \| Age, years, median (IQR) \| 43 \| 28-55 \| 36 \| 29-46 \| <0.001 \| \| BMI, median, (IQR) \| 26.6 \| 23.2-31.0 \| 26.0 \| 23.5-28.7 \| <0.001 \| \| Cold Ischemia time, hours, median, (IQR) \| 6.1 \| 4.9-8.0 \| 1.5 \| 1.0-2.1 \| <0.001 \|   **Legend**: LDLT=live donor liver transplant, DDLT=deceased donor liver transplant, MELD=model for end stage liver disease, BMI=body mass index   \| **Supplementary Table 2C:** Recipient and donor characteristics of patients transplanted with MELD score 21-25 before propensity score matching. \| \| \| \| \| \| \| --- \| --- \| --- \| --- \| --- \| --- \| \| **Characteristics** \| **DDLT** \| \| **LDLT** \| \| **P Value** \| \| ***N. of patients*** \| 12,861 \| \| 386 \| \| \| Female sex, n. (%) \| 4,503 \| 35.0% \| 174 \| 45.1% \| <0.001 \| \| Age, years, median (IQR) \| 55.0 \| 49-62 \| 54.0 \| 44-62 \| <0.001 \| \| **Etiology of liver disease, n. (%)** \|  \|  \|  \|  \| <0.001 \| \| Alcohol \| 3,490 \| 27.1% \| 75 \| 19.4% \| \| Nonalcoholic fatty liver disease \| 1,807 \| 14.1% \| 68 \| 17.6% \| \| Primary biliary cirrhosis / Primary sclerosing cholangitis \| 1,446 \| 11.2% \| 115 \| 29.8% \| \| Hepatocellular carcinoma \| 970 \| 7.5% \| 14 \| 3.6% \| \| Viral \| 2,723 \| 21.2% \| 55 \| 14.2% \| \| Other \| 2,425 \| 18.8% \| 59 \| 15.2% \| \| **Race, n. (%)** \|  \|  \|  \|  \| <0.001 \| \| White or Hispanic \| 11,047 \| 85.9% \| 360 \| 93.3% \| \| Black \| 1,316 \| 10.2% \| 18 \| 4.7% \| \| Asian \| 336 \| 2.6% \| 5 \| 1.3% \| \| Other/unknown \| 162 \| 1.3% \| 3 \| 0.8% \| \| **Highest level of Education, n. (%)** \|  \|  \|  \|  \| <0.001 \| \| Unknown \| 1,454 \| 11.3% \| 55 \| 14.2% \| \| Grade 1-8 \| 524 \| 4.1% \| 11 \| 2.8% \| \| High school Degree \| 4,980 \| 38.7% \| 130 \| 33.7% \| \| College-University Degree \| 5,903 \| 45.9% \| 190 \| 49.2% \| \| **Primary Payer, n. (%)** \|  \|  \|  \|  \| <0.001 \| \| Private health insurance \| 8,825 \| 54.3% \| 329 \| 70.0% \| \| Medicare/Medicaid \| 6,530 \| 40.2% \| 135 \| 28.7% \| \| Public health insurance \| 559 \| 3.4% \| 1 \| 0.2% \| \| Other payers \| 338 \| 2.1% \| 5 \| 1.1% \| \| **Recipient blood group, n. (%)** \|  \|  \|  \|  \| 0.002 \| \| O \| 5,743 \| 44.7% \| 121 \| 46.9% \| \| A \| 4,831 \| 37.6% \| 166 \| 43.0% \| \| B \| 1,659 \| 12.9% \| 33 \| 8.5% \| \| AB \| 628 \| 4.9% \| 6 \| 1.6% \| \| BMI, median, IQR \| 27.7 \| 24.1-32.0 \| 26.6 \| 23.5-30.7 \| 0.003 \| \| Dialysis, n. (%) \| 2,677 \| 16.50% \| 8 \| 1.70% \| <0.001 \| \| Diabetes, n. (%) \| 4,349 \| 26.8% \| 78 \| 16.6% \| 0.002 \| \| TIPS, n. (%) \| 1,344 \| 10.5% \| 28 \| 7.3% \| 0.091 \| \| Portal vein thrombosis, n. (%) \| 1,501 \| 11.7% \| 25 \| 6.5% \| <0.001 \| \| Life support, n. (%) \| 1,002 \| 7.8% \| 6 \| 1.3% \| <0.001 \| \| Ventilator, n. (%) \| 625 \| 4.9% \| 0 \| 0.0% \| <0.001 \| \| Spontaneous Bacterial Peritonitis, n. (%) \| 725 \| 5.6% \| 13 \| 3.4% \| 0.024 \| \| Prior abdominal surgery, n. (%) \| 9,178 \| 56.5% \| 281 \| 59.8% \| 0.153 \| \| **Donor Characteristics** \|  \|  \|  \|  \|  \| \| Female sex, n. (%) \| 6,512 \| 40.1% \| 249 \| 53.0% \| <0.001 \| \| Age, years, median (IQR) \| 41 \| 27-54 \| 38 \| 30-47 \| <0.001 \| \| BMI, median, (IQR) \| 26.45 \| 23.1-30.9 \| 26.93 \| 24.3-29.2 \| <0.001 \| \| Cold Ischemia time, hours, median, (IQR) \| 6.1 \| 4.9-7.9 \| 1.7 \| 1.0-2.3 \| <0.001 \|   **Legend**: LDLT=live donor liver transplant, DDLT=deceased donor liver transplant, MELD=model for end stage liver disease, BMI=body mass index   \| **Supplementary Table 2D:** Recipient and donor characteristics of patients transplanted with MELD score 26-30 before propensity score matching. \| \| \| \| \| \| \| --- \| --- \| --- \| --- \| --- \| --- \| \| **Characteristics** \| **DDLT** \| \| **LDLT** \| \| **P Value** \| \| ***N. of patients*** \| 11,603 \| \| 107 \| \| \| Female sex, n. (%) \| 4,505 \| 38.8% \| 43 \| 40.2% \| 0.475 \| \| Age, years, median (IQR) \| 54 \| 47-61 \| 56 \| 47-62 \| 0.093 \| \| **Etiology of liver disease, n. (%)** \|  \|  \|  \|  \| <0.001 \| \| Alcohol \| 3,532 \| 30.4% \| 19 \| 17.8% \| \| Nonalcoholic fatty liver disease \| 1,537 \| 13.2% \| 27 \| 25.2% \| \| Primary biliary cirrhosis / Primary sclerosing cholangitis \| 1,159 \| 10.0% \| 19 \| 17.8% \| \| Hepatocellular carcinoma \| 576 \| 5.0% \| 6 \| 5.6% \| \| Viral \| 2,237 \| 19.3% \| 13 \| 12.1% \| \| Other \| 2,562 \| 22.0% \| 23 \| 21.4% \| \| **Race, n. (%)** \|  \|  \|  \|  \| 0.002 \| \| White or Hispanic \| 9,835 \| 84.8% \| 102 \| 95.3% \| \| Black \| 1,203 \| 10.4% \| 2 \| 1.9% \| \| Asian \| 382 \| 3.3% \| 3 \| 2.8% \| \| Other/unknown \| 183 \| 1.6% \| 0 \| 0.0% \| \| **Highest level of Education, n. (%)** \|  \|  \|  \|  \| 0.008 \| \| Unknown \| 1,412 \| 10.9% \| 21 \| 19.6% \| \| Grade 1-8 \| 616 \| 4.7% \| 3 \| 2.8% \| \| High school Degree \| 5,072 \| 39.1% \| 30 \| 28.0% \| \| College-University Degree \| 5,874 \| 45.3% \| 53 \| 49.5% \| \| **Primary Payer, n. (%)** \|  \|  \|  \|  \| <0.001 \| \| Private health insurance \| 7,092 \| 54.7% \| 96 \| 71.6% \| \| Medicare/Medicaid \| 5,189 \| 40.0% \| 36 \| 26.9% \| \| Public health insurance \| 462 \| 3.6% \| 1 \| 0.7% \| \| Other payers \| 231 \| 1.8% \| 1 \| 0.7% \| \| **Recipient blood group, n. (%)** \|  \|  \|  \|  \| 0.006 \| \| O \| 5,387 \| 46.4% \| 57 \| 53.3% \| \| A \| 4,346 \| 37.5% \| 46 \| 43.0% \| \| B \| 1,423 \| 12.3% \| 4 \| 3.7% \| \| AB \| 447 \| 3.9% \| 0 \| 0.0% \| \| BMI, median, IQR \| 27.6 \| 24.0-32.2 \| 27.0 \| 23.0-31.7 \| 0.642 \| \| Dialysis, n. (%) \| 3,322 \| 25.6% \| 5 \| 3.7% \| <0.001 \| \| Diabetes, n. (%) \| 3,169 \| 24.4% \| 21 \| 15.7% \| 0.006 \| \| TIPS, n. (%) \| 985 \| 8.5% \| 2 \| 1.9% \| 0.045 \| \| Portal vein thrombosis, n. (%) \| 1,256 \| 10.8% \| 2 \| 1.9% \| 0.017 \| \| Life support, n. (%) \| 1,863 \| 16.1% \| 2 \| 1.9% \| <0.001 \| \| Ventilator, n. (%) \| 1,217 \| 10.5% \| 2 \| 1.9% \| <0.001 \| \| Spontaneous Bacterial Peritonitis, n. (%) \| 803 \| 6.9% \| 2 \| 1.9% \| 0.036 \| \| Prior abdominal surgery, n. (%) \| 7,547 \| 58.2% \| 80 \| 59.7% \| 0.721 \| \| **Donor Characteristics** \|  \|  \|  \|  \|  \| \| Female Sex , n. (%) \| 5,247 \| 40.4% \| 74 \| 55.2% \| <0.001 \| \| Age, years, median (IQR) \| 40 \| 26-52 \| 37 \| 29.46 \| <0.001 \| \| BMI, median, (IQR) \| 26.37 \| 23.1-30.3 \| 26.45 \| 23.6-29.5 \| <0.001 \| \| Cold Ischemia time, hours, median, (IQR) \| 6.3 \| 4.9-7.8 \| 2 \| 1.0-2.4 \| <0.001 \|   **Legend**: LDLT=live donor liver transplant, DDLT=deceased donor liver transplant, MELD=model for end stage liver disease, BMI=body mass index   \| **Supplementary Table 2E:** Recipient and donor characteristics of patients transplanted with MELD score >30 before propensity score matching. \| \| \| \| \| \| \| --- \| --- \| --- \| --- \| --- \| --- \| \| **Characteristics** \| **DDLT** \| \| **LDLT** \| \| **P Value** \| \| ***N. of patients*** \| 30,638 \| \| 466 \| \|  \| \| Female sex, n. (%) \| 11,015 \| 36.0% \| 202 \| 43.3% \| 0.004 \| \| Age, years, median (IQR) \| 53 \| 45-60 \| 57 \| 35-66 \|  \| \| **Etiology of liver disease, n. (%)** \|  \|  \|  \|  \| 0.014 \| \| Alcohol \| 8,481 \| 27.7% \| 78 \| 16.7% \| \| Nonalcoholic fatty liver disease \| 3,438 \| 11.2% \| 66 \| 14.2% \| \| Primary biliary cirrhosis / Primary sclerosing cholangitis \| 2,846 \| 9.3% \| 118 \| 25.3% \| \| Hepatocellular carcinoma \| 2,157 \| 7.0% \| 29 \| 6.2% \| \| Viral \| 6,684 \| 21.8% \| 85 \| 18.2% \| \| Other \| 7,030 \| 22.9% \| 90 \| 19.3% \| \| **Race, n. (%)** \|  \|  \|  \|  \| 0.044 \| \| White or Hispanic \| 25,695 \| 83.9% \| 431 \| 92.5% \| \| Black \| 3,239 \| 10.9% \| 18 \| 3.9% \| \| Asian \| 1,123 \| 3.7% \| 14 \| 3.0% \| \| Other/unknown \| 491 \| 1.6% \| 3 \| 0.6% \| \| **Highest level of Education, n. (%)** \|  \|  \|  \|  \| 0.436 \| \| Unknown \| 3,433 \| 11.2 \| 49 \| 10.5% \| \| Grade 1-8 \| 1,341 \| 4.4 \| 10 \| 2.1% \| \| High school Degree \| 12,089 \| 39.5% \| 140 \| 30.0% \| \| College-University Degree \| 13,775 \| 45.0% \| 267 \| 57.3% \| \| **Primary Payer, n. (%)** \|  \|  \|  \|  \| 0.935 \| \| Private health insurance \| 11,329 \| 56.1% \| 27 \| 60.0% \| \| Medicare/Medicaid \| 7,842 \| 38.8% \| 16 \| 35.6% \| \| Public health insurance \| 651 \| 3.2% \| 1 \| 2.2% \| \| Other payers \| 369 \| 1.8% \| 1 \| 2.2% \| \| **Recipient blood group, n. (%)** \|  \|  \|  \|  \| 0.627 \| \| O \| 14,132 \| 46.1% \| 230 \| 49.4% \| \| A \| 11,235 \| 36.7% \| 176 \| 37.8% \| \| B \| 3,991 \| 13.0% \| 52 \| 11.2% \| \| AB \| 1,280 \| 4.2% \| 8 \| 1.7% \| \| BMI, median, IQR \| 28.2 \| 24.4-32.9 \| 25.9 \| 23.5-31.7 \| 0.002 \| \| Dialysis, n. (%) \| 6,701 \| 33.2% \| 4 \| 8.9% \| <0.001 \| \| Diabetes, n. (%) \| 4,438 \| 22.0% \| 11 \| 24.4% \| 0.69 \| \| TIPS, n. (%) \| 2,787 \| 9.1% \| 40 \| 8.6% \| 0.163 \| \| Portal vein thrombosis, n. (%) \| 3,288 \| 10.7% \| 47 \| 10.1% \| 0.427 \| \| Life support, n. (%) \| 5,029 \| 16.4% \| 6 \| 1.3% \| 0.032 \| \| Ventilator, n. (%) \| 3,228 \| 10.5% \| 6 \| 1.3% \| 0.312 \| \| Spontaneous Bacterial Peritonitis, n. (%) \| 1,980 \| 6.5% \| 13 \| 2.8% \| 0.446 \| \| Prior abdominal surgery, n. (%) \| 12,339 \| 61.1% \| 28 \| 62.2% \| 0.879 \| \| **Donor Characteristics** \|  \|  \|  \|  \|  \| \| Female sex, n. (%) \| 7,952 \| 39.4% \| 27 \| 60.0% \| 0.005 \| \| Age, years, median (IQR) \| 38 \| 25-51 \| 36 \| 27-49 \| 0.004 \| \| BMI, median, (IQR) \| 26.05 \| 23-30.0 \| 26.55 \| 23.0-28.5 \| 0.002 \| \| Cold Ischemia time, hours, median, (IQR) \| 6.1 \| 5.0-7.9 \| 1.7 \| 1.0-2.2 \| <0.001 \|   **Legend**: LDLT=live donor liver transplant, DDLT=deceased donor liver transplant, MELD=model for end stage liver disease, BMI=body mass index  **Supplementary Table 3:** Primary cause of death between recipients of live donor liver transplant (LDLT) and deceased donor liver transplant (DDLT) before propensity score matching.   \| **Cause of Death** \| **DDLT** \| \| **LDLT** \| \| **P Value** \| \| --- \| --- \| --- \| --- \| --- \| --- \| \| **Count** \| **Percentage** \| **Count** \| **Percentage** \| \| Cardiovascular \| 3,656 \| 11.2 \| 82 \| 9.5 \| 0.066 \| \| Cerebrovascular \| 719 \| 2.3 \| 20 \| 2.3 \| 0.919 \| \| Respiratory \| 1,522 \| 4.8 \| 51 \| 5.9 \| 0.132 \| \| Graft failure \| 3,143 \| 9.9 \| 66 \| 7.6 \| **0.035** \| \| Infection \| 6,544 \| 20.6 \| 187 \| 21.6 \| 0454 \| \| Malignancy \| 2,351 \| 7.4 \| 72 \| 8.3 \| 0.302 \| \| Hemorrhage \| 918 \| 2.9 \| 35 \| 4.1 \| **0.045** \| \| Unknown \| 5,880 \| 18.5 \| 148 \| 17.1 \| 0.302 \| \| Other \| 7,035 \| 22.1 \| 203 \| 23.5 \| 0.345 \|   **Supplementary Table 4:** Fully adjusted hazard ratio (aHR) for the risk of patients’ death after liver transplantation before propensity score matching.   \| **Variable** \| **Adjusted Hazard Ratio (aHR)** \| **95.0% Confidence Interval** \| \| **P Value** \| \| --- \| --- \| --- \| --- \| --- \| \| **Lower** \| **Upper** \| \| Live donor liver transplantation \| **1 (Reference)** \|  \|  \| 0.263 \| \| Deceased donor liver transplantation \| 1.07 \| 0.95 \| 1.20 \| \| **Sex** \|  \|  \|  \|  \| \| Male \| **1 (Reference)** \|  \|  \|  \| \| Female \| 0.909 \| 0.88 \| 0.94 \| <.001 \| \| **Age** \|  \|  \|  \| <.001 \| \| ≤50 \| **1 (Reference)** \|  \|  \|  \| \| 51-64 \| 1.18 \| 1.14 \| 1.22 \| <.001 \| \| ≥65 \| 1.45 \| 1.38 \| 1.51 \| <.001 \| \| **Indication for liver transplantation** \|  \|  \|  \| <.001 \| \| Alcohol \| **1 (Reference)** \|  \|  \|  \| \| Acute \| 0.96 \| 0.89 \| 1.04 \| 0.326 \| \| Nonalcoholic fatty liver disease \| 1.03 \| 0.97 \| 1.09 \| 0.395 \| \| Autoimmune disease \| 0.81 \| 0.76 \| 0.86 \| <.001 \| \| Hepatocellular carcinoma \| 1.38 \| 1.31 \| 1.44 \| <.001 \| \| Viral \| 1.23 \| 1.18 \| 1.28 \| <.001 \| \| Other \| 1.09 \| 1.04 \| 1.14 \| <.001 \| \| **Race** \|  \|  \|  \| <.001 \| \| White/Caucasian \| **1 (Reference)** \|  \|  \|  \| \| Black/African American \| 1.29 \| 1.24 \| 1.35 \| <.001 \| \| Asian \| 0.76 \| 0.70 \| 0.82 \| <.001 \| \| Other / Unknown \| 1.03 \| 0.91 \| 1.16 \| 0.656 \| \| **MELD** \|  \|  \|  \| <.001 \| \| <15 \| **1 (Reference)** \|  \|  \|  \| \| 16-20 \| 0.93 \| 0.89 \| 0.97 \| <.001 \| \| 21-25 \| 1.01 \| 0.97 \| 0.06 \| 0.377 \| \| 26-30 \| 1.09 \| 1.04 \| 0.14 \| <0.01 \| \| >30 \| 1.20 \| 1.15 \| 1.24 \| <.001 \| \| **Performance status, Karnofsky score** \| 0.99 \| 0.99 \| 0.99 \| <.001 \| \| **Education** \|  \|  \|  \| <.001 \| \| No education / unknown \| **1 (Reference)** \|  \|  \|  \| \| Elementary School (Grade 1-8) \| 0.92 \| 0.85 \| 0.99 \| 0.023 \| \| High School (Grade 9-12) \| 0.97 \| 0.93 \| 1.01 \| 0.140 \| \| College / University degree \| 0.92 \| 0.88 \| 0.96 \| <.001 \| \| **Payment source** \|  \|  \|  \| <.001 \| \| Private insurance \| **1 (Reference)** \|  \|  \|  \| \| Medicare/Medicaid \| 1.18 \| 1.15 \| 1.22 \| <.001 \| \| Public insurance \| 1.06 \| 0.99 \| 1.15 \| 0.111 \| \| Other \| 1.30 \| 1.20 \| 1.41 \| <.001 \| \| **BMI** \|  \|  \|  \| <.001 \| \| 18.5-24.9 \| **1 (Reference)** \|  \|  \|  \| \| <18.5 \| 1.23 \| 1.12 \| 1.34 \| <.001 \| \| 25-29.9 \| 0.89 \| 0.86 \| 0.92 \| <.001 \| \| >30 \| 0.88 \| 0.85 \| 0.91 \| <.001 \| \| **Dialysis** \| 1.17 \| 1.13 \| 1.23 \| <.001 \| \| **Diabetes** \| 1.20 \| 1.17 \| 1.24 \| <.001 \| \| **Portal vein thrombosis** \| 1.18 \| 1.13 \| 1.24 \| <.001 \| \| **Ventilator support** \| 1.51 \| 1.38 \| 1.65 \| <.001 \| \| **Spontaneous bacterial peritonitis** \| 1.06 \| 1.00 \| 1.12 \| 0.049 \| \| **Trans jugular intrahepatic portosystemic shunt** \| 1.04 \| 1.00 \| 1.09 \| 0.067 \| \| **Prior abdominal surgery** \| 1.17 \| 1.14 \| 1.20 \| <.001 \| \| **Life support** \| 1.11 \| 1.02 \| 1.20 \| 0.017 \| \| **Donor age, years** \|  \|  \|  \| <.001 \| \| ≤50 \| **1 (Reference)** \|  \|  \|  \| \| 51-64 \| 1.21 \| 1.17 \| 1.24 \| <.001 \| \| ≥65 \| 1.38 \| 1.32 \| 1.45 \| <.001 \| \| **Donor BMI** \|  \|  \|  \| 0.013 \| \| 18.5-24.9 \| **1 (Reference)** \|  \|  \|  \| \| <18.5 \| 1.05 \| 0.97 \| 1.13 \| 0.263 \| \| 25-29.9 \| 1.00 \| 0.97 \| 1.03 \| 0.920 \| \| >30 \| 1.05 \| 1.02 \| 1.09 \| 0.004 \| \| **Year of transplantation** \| 0.94 \| 0.94 \| 0.94 \| <0.001 \| \| **Cold ischemia time, hours** \|  \|  \|  \| <0.001 \| \| <6 \| **1 (Reference)** \|  \|  \|  \| \| 6 - 12 \| 1.10 \| 1.07 \| 1.13 \| <.001 \| \| >12h \| 1.29 \| 1.20 \| 1.39 \| <.001 \| \|  \|  \|  \|  \|  \|   **Legend:** BMI = Body Mass Index, MELD=Model for End Stage Liver Disease, aHR=adjusted hazard ratio |  |
| --- | --- | --- | --- | --- | --- | --- | --- | --- | --- | --- | --- | --- | --- | --- | --- | --- | --- | --- | --- | --- | --- | --- | --- | --- | --- | --- | --- | --- | --- | --- | --- | --- | --- | --- | --- | --- | --- | --- | --- | --- | --- | --- | --- | --- | --- | --- | --- | --- | --- | --- | --- | --- | --- | --- | --- | --- | --- | --- | --- | --- | --- | --- | --- | --- | --- | --- | --- | --- | --- | --- | --- | --- | --- | --- | --- | --- | --- | --- | --- | --- | --- | --- | --- | --- | --- | --- | --- | --- | --- | --- | --- | --- | --- | --- | --- | --- | --- | --- | --- | --- | --- | --- | --- | --- | --- | --- | --- | --- | --- | --- | --- | --- | --- | --- | --- | --- | --- | --- | --- | --- | --- | --- | --- | --- | --- | --- | --- | --- | --- | --- | --- | --- | --- | --- | --- | --- | --- | --- | --- | --- | --- | --- | --- | --- | --- | --- | --- | --- | --- | --- | --- | --- | --- | --- | --- | --- | --- | --- | --- | --- | --- | --- | --- | --- | --- | --- | --- | --- | --- | --- | --- | --- | --- | --- | --- | --- | --- | --- | --- | --- | --- | --- | --- | --- | --- | --- | --- | --- | --- | --- | --- | --- | --- | --- | --- | --- | --- | --- | --- | --- | --- | --- | --- | --- | --- | --- | --- | --- | --- | --- | --- | --- | --- | --- | --- | --- | --- | --- | --- | --- | --- | --- | --- | --- | --- | --- | --- | --- | --- | --- | --- | --- | --- | --- | --- | --- | --- | --- | --- | --- | --- | --- | --- | --- | --- | --- | --- | --- | --- | --- | --- | --- | --- | --- | --- | --- | --- | --- | --- | --- | --- | --- | --- | --- | --- | --- | --- | --- | --- | --- | --- | --- | --- | --- | --- | --- | --- | --- | --- | --- | --- | --- | --- | --- | --- | --- | --- | --- | --- | --- | --- | --- | --- | --- | --- | --- | --- | --- | --- | --- | --- | --- | --- | --- | --- | --- | --- | --- | --- | --- | --- | --- | --- | --- | --- | --- | --- | --- | --- | --- | --- | --- | --- | --- | --- | --- | --- | --- | --- | --- | --- | --- | --- | --- | --- | --- | --- | --- | --- | --- | --- | --- | --- | --- | --- | --- | --- | --- | --- | --- | --- | --- | --- | --- | --- | --- | --- | --- | --- | --- | --- | --- | --- | --- | --- | --- | --- | --- | --- | --- | --- | --- | --- | --- | --- | --- | --- | --- | --- | --- | --- | --- | --- | --- | --- | --- | --- | --- | --- | --- | --- | --- | --- | --- | --- | --- | --- | --- | --- | --- | --- | --- | --- | --- | --- | --- | --- | --- | --- | --- | --- | --- | --- | --- | --- | --- | --- | --- | --- | --- | --- | --- | --- | --- | --- | --- | --- | --- | --- | --- | --- | --- | --- | --- | --- | --- | --- | --- | --- | --- | --- | --- | --- | --- | --- | --- | --- | --- | --- | --- | --- | --- | --- | --- | --- | --- | --- | --- | --- | --- | --- | --- | --- | --- | --- | --- | --- | --- | --- | --- | --- | --- | --- | --- | --- | --- | --- | --- | --- | --- | --- | --- | --- | --- | --- | --- | --- | --- | --- | --- | --- | --- | --- | --- | --- | --- | --- | --- | --- | --- | --- | --- | --- | --- | --- | --- | --- | --- | --- | --- | --- | --- | --- | --- | --- | --- | --- | --- | --- | --- | --- | --- | --- | --- | --- | --- | --- | --- | --- | --- | --- | --- | --- | --- | --- | --- | --- | --- | --- | --- | --- | --- | --- | --- | --- | --- | --- | --- | --- | --- | --- | --- | --- | --- | --- | --- | --- | --- | --- | --- | --- | --- | --- | --- | --- | --- | --- | --- | --- | --- | --- | --- | --- | --- | --- | --- | --- | --- | --- | --- | --- | --- | --- | --- | --- | --- | --- | --- | --- | --- | --- | --- | --- | --- | --- | --- | --- | --- | --- | --- | --- | --- | --- | --- | --- | --- | --- | --- | --- | --- | --- | --- | --- | --- | --- | --- | --- | --- | --- | --- | --- | --- | --- | --- | --- | --- | --- | --- | --- | --- | --- | --- | --- | --- | --- | --- | --- | --- | --- | --- | --- | --- | --- | --- | --- | --- | --- | --- | --- | --- | --- | --- | --- | --- | --- | --- | --- | --- | --- | --- | --- | --- | --- | --- | --- | --- | --- | --- | --- | --- | --- | --- | --- | --- | --- | --- | --- | --- | --- | --- | --- | --- | --- | --- | --- | --- | --- | --- | --- | --- | --- | --- | --- | --- | --- | --- | --- | --- | --- | --- | --- | --- | --- | --- | --- | --- | --- | --- | --- | --- | --- | --- | --- | --- | --- | --- | --- | --- | --- | --- | --- | --- | --- | --- | --- | --- | --- | --- | --- | --- | --- | --- | --- | --- | --- | --- | --- | --- | --- | --- | --- | --- | --- | --- | --- | --- | --- | --- | --- | --- | --- | --- | --- | --- | --- | --- | --- | --- | --- | --- | --- | --- | --- | --- | --- | --- | --- | --- | --- | --- | --- | --- | --- | --- | --- | --- | --- | --- | --- | --- | --- | --- | --- | --- | --- | --- | --- | --- | --- | --- | --- | --- | --- | --- | --- | --- | --- | --- | --- | --- | --- | --- | --- | --- | --- | --- | --- | --- | --- | --- | --- | --- | --- | --- | --- | --- | --- | --- | --- | --- | --- | --- | --- | --- | --- | --- | --- | --- | --- | --- | --- | --- | --- | --- | --- | --- | --- | --- | --- | --- | --- | --- | --- | --- | --- | --- | --- | --- | --- | --- | --- | --- | --- | --- | --- | --- | --- | --- | --- | --- | --- | --- | --- | --- | --- | --- | --- | --- | --- | --- | --- | --- | --- | --- | --- | --- | --- | --- | --- | --- | --- | --- | --- | --- | --- | --- | --- | --- | --- | --- | --- | --- | --- | --- | --- | --- | --- | --- | --- | --- | --- | --- | --- | --- | --- | --- | --- | --- | --- | --- | --- | --- | --- | --- | --- | --- | --- | --- | --- | --- | --- | --- | --- | --- | --- | --- | --- | --- | --- | --- | --- | --- | --- | --- | --- | --- | --- | --- | --- | --- | --- | --- | --- | --- | --- | --- | --- | --- | --- | --- | --- | --- | --- | --- | --- | --- | --- | --- | --- | --- | --- | --- | --- | --- | --- | --- | --- | --- | --- | --- | --- | --- | --- | --- | --- | --- | --- | --- | --- | --- | --- | --- | --- | --- | --- | --- | --- | --- | --- | --- | --- | --- | --- | --- | --- | --- | --- | --- | --- | --- | --- | --- | --- | --- | --- | --- | --- | --- | --- | --- | --- | --- | --- | --- | --- | --- | --- | --- | --- | --- | --- | --- | --- | --- | --- | --- | --- | --- | --- | --- | --- | --- | --- | --- | --- | --- | --- | --- | --- | --- | --- | --- | --- | --- | --- | --- | --- | --- | --- | --- | --- | --- | --- | --- | --- | --- | --- | --- | --- | --- | --- | --- | --- | --- | --- | --- | --- | --- | --- | --- | --- | --- | --- | --- | --- | --- | --- | --- | --- | --- | --- | --- | --- | --- | --- | --- | --- | --- | --- | --- | --- | --- | --- | --- | --- | --- | --- | --- | --- | --- | --- | --- | --- | --- | --- | --- | --- | --- | --- | --- | --- | --- | --- | --- | --- | --- | --- | --- | --- | --- | --- | --- | --- | --- | --- | --- | --- | --- | --- | --- | --- | --- | --- | --- | --- | --- | --- | --- | --- | --- | --- | --- | --- | --- | --- | --- | --- | --- | --- | --- | --- | --- | --- | --- | --- | --- | --- | --- | --- | --- | --- | --- | --- | --- | --- | --- | --- | --- | --- | --- | --- | --- | --- | --- | --- | --- | --- | --- | --- | --- | --- | --- | --- | --- | --- | --- | --- | --- | --- | --- | --- | --- | --- | --- | --- | --- | --- | --- | --- | --- | --- | --- | --- | --- | --- | --- | --- | --- | --- | --- | --- | --- | --- | --- | --- | --- | --- | --- | --- | --- | --- | --- | --- | --- | --- | --- | --- | --- | --- | --- | --- | --- | --- | --- | --- | --- | --- | --- | --- | --- | --- | --- | --- | --- | --- | --- | --- | --- | --- | --- | --- | --- | --- | --- | --- | --- | --- | --- | --- | --- | --- | --- | --- | --- | --- | --- | --- | --- | --- | --- | --- | --- | --- | --- | --- | --- | --- | --- | --- | --- | --- | --- | --- | --- | --- | --- | --- | --- | --- | --- | --- | --- | --- | --- | --- | --- | --- | --- | --- | --- | --- | --- | --- | --- | --- | --- | --- | --- | --- | --- | --- | --- | --- | --- | --- | --- | --- | --- | --- | --- | --- | --- | --- | --- | --- | --- | --- | --- | --- | --- | --- | --- | --- | --- | --- | --- | --- | --- | --- | --- | --- | --- | --- | --- | --- | --- | --- | --- | --- | --- | --- | --- | --- | --- | --- | --- | --- | --- | --- | --- | --- | --- | --- | --- | --- | --- | --- | --- | --- | --- | --- | --- | --- | --- | --- | --- | --- | --- | --- | --- | --- | --- | --- | --- | --- | --- | --- | --- | --- | --- | --- | --- | --- | --- | --- | --- | --- | --- | --- | --- | --- | --- | --- | --- | --- | --- | --- | --- | --- | --- | --- | --- | --- | --- | --- | --- | --- | --- | --- | --- | --- | --- | --- | --- | --- | --- | --- | --- | --- | --- | --- | --- | --- | --- | --- | --- | --- | --- | --- | --- | --- | --- | --- | --- | --- | --- | --- | --- | --- | --- | --- | --- | --- | --- | --- | --- | --- | --- | --- | --- | --- | --- | --- | --- | --- | --- | --- | --- | --- | --- | --- | --- | --- | --- | --- | --- | --- | --- | --- | --- | --- | --- | --- | --- | --- | --- | --- | --- | --- | --- | --- | --- | --- | --- | --- | --- | --- | --- | --- | --- | --- | --- | --- | --- | --- | --- | --- | --- | --- | --- | --- | --- | --- | --- | --- | --- | --- | --- | --- | --- | --- | --- | --- | --- | --- | --- | --- | --- | --- | --- | --- | --- | --- | --- | --- | --- | --- | --- | --- | --- | --- | --- | --- | --- | --- | --- | --- | --- | --- | --- | --- | --- | --- | --- | --- | --- | --- | --- | --- | --- | --- | --- | --- | --- | --- | --- | --- | --- | --- | --- | --- | --- | --- | --- | --- | --- | --- | --- | --- | --- | --- | --- | --- | --- | --- | --- | --- | --- | --- | --- | --- | --- | --- | --- | --- | --- | --- | --- | --- | --- | --- | --- | --- | --- | --- | --- | --- | --- | --- | --- | --- | --- | --- | --- | --- | --- | --- | --- | --- | --- | --- | --- | --- | --- | --- | --- | --- | --- | --- | --- | --- | --- | --- | --- | --- | --- | --- | --- | --- | --- | --- | --- | --- | --- | --- | --- | --- | --- | --- | --- | --- | --- | --- | --- | --- | --- | --- | --- | --- | --- | --- | --- | --- | --- | --- | --- | --- | --- | --- | --- | --- | --- | --- | --- | --- | --- | --- | --- | --- | --- | --- | --- | --- | --- | --- | --- | --- | --- | --- | --- | --- | --- | --- | --- | --- | --- | --- | --- | --- | --- | --- | --- | --- | --- | --- | --- | --- | --- | --- | --- | --- | --- | --- | --- | --- | --- | --- | --- | --- | --- | --- | --- | --- | --- | --- | --- | --- | --- | --- | --- | --- | --- | --- | --- | --- | --- | --- | --- | --- | --- | --- | --- | --- | --- | --- | --- | --- | --- | --- | --- | --- | --- | --- | --- | --- | --- | --- | --- | --- | --- | --- | --- | --- | --- | --- | --- | --- | --- | --- | --- | --- | --- | --- | --- | --- | --- | --- | --- | --- | --- | --- | --- | --- | --- | --- | --- | --- | --- | --- | --- | --- | --- | --- | --- | --- | --- | --- | --- | --- | --- | --- | --- | --- | --- | --- | --- | --- | --- | --- | --- | --- | --- | --- | --- | --- | --- | --- | --- | --- | --- | --- | --- | --- | --- | --- | --- | --- | --- | --- | --- | --- | --- | --- | --- | --- | --- | --- | --- | --- | --- | --- | --- | --- | --- | --- | --- | --- | --- | --- | --- | --- | --- | --- | --- | --- | --- | --- | --- | --- | --- | --- | --- | --- | --- | --- | --- | --- | --- | --- | --- | --- | --- | --- | --- | --- | --- | --- | --- | --- | --- | --- | --- | --- | --- | --- | --- | --- | --- | --- | --- | --- | --- | --- | --- | --- | --- | --- | --- | --- | --- | --- | --- | --- | --- | --- | --- | --- | --- | --- | --- | --- | --- | --- | --- | --- | --- | --- | --- | --- | --- | --- | --- | --- | --- | --- | --- | --- | --- | --- | --- | --- | --- | --- | --- | --- | --- | --- | --- | --- | --- | --- | --- | --- | --- | --- | --- | --- | --- | --- | --- | --- | --- | --- | --- | --- | --- | --- | --- | --- | --- | --- | --- | --- | --- | --- | --- | --- | --- | --- | --- | --- | --- | --- | --- | --- | --- | --- | --- | --- | --- | --- | --- | --- | --- | --- | --- | --- | --- | --- | --- | --- | --- | --- | --- | --- | --- | --- | --- | --- | --- | --- | --- | --- | --- | --- | --- | --- | --- | --- | --- | --- | --- | --- | --- | --- | --- | --- | --- | --- | --- | --- | --- | --- | --- | --- | --- | --- | --- | --- | --- | --- | --- | --- | --- | --- | --- | --- | --- | --- | --- | --- | --- | --- | --- | --- | --- | --- | --- | --- | --- | --- | --- | --- | --- | --- | --- | --- | --- | --- | --- | --- | --- | --- | --- | --- | --- | --- | --- | --- | --- | --- | --- | --- | --- | --- | --- | --- | --- | --- | --- | --- | --- | --- | --- | --- | --- | --- | --- | --- | --- | --- | --- | --- | --- | --- | --- | --- | --- | --- | --- | --- | --- | --- | --- | --- | --- | --- | --- | --- | --- | --- | --- | --- | --- | --- | --- | --- | --- | --- | --- | --- | --- | --- | --- | --- | --- | --- | --- | --- | --- | --- | --- | --- | --- | --- | --- | --- | --- | --- | --- | --- | --- | --- | --- | --- | --- | --- |

| **Supplementary Table 5A.**  Standardized mean difference and standard error of the difference (SED) before and after propensity score matching between recipients of live donor liver transplantation (LDLT) versus recipients of deceased donor liver transplantation (DDLT) with MELD <15. | | | | | | | |  |
| --- | --- | --- | --- | --- | --- | --- | --- | --- |
|  |  |  |  |  |  |  |  |  |
| **MELD <15** | **Before Propensity Score Matching** | | | **After Propensity Score Matching** | | |  |  |
| **Characteristics** | LDLT, n. 2,162 | DDLT, n. 31,174 | **P Value** | LDLT, n. 2,038 | DDLT, n. 15,537 | **P Value** |  |  |
|  | **Standardized Mean Difference (SED)** | |  | **Standardized Mean Difference (SED)** | |  |  |  |
|  |  |  |  |  |  |  |  | |
| Recipient sex | **0.175 (0.010)** | | **<0.001** | **0.076 (0.011)** | | **<0.001** |  | |
| Recipient age | **4.232 (0.217)** | | **<0.001** | **2.007 (0.242)** | | **<0.001** |  | |
| Recipient race/ethnicity | **0.119 (0.135)** | | **<0.001** | **0.026 (0.011)** | | **0.008** |  | |
| Recipient body mass index | **0.192 (0.027)** | | **<0.001** | **0.679 (0.122)** | | **<0.001** |  | |
| Recipient highest degree of education | **0.111 (0.021)** | | **<0.001** | **0.044 (0.021)** | | **0.034** |  | |
| Recipient functional status | **1.671 (0.406)** | | **<0.001** | 0.443 (0.438) | | **0.311** |  | |
| Recipient type of healthcare insurance | **0.194 (0.015)** | | **<0.001** | **0.055 (0.013)** | | **<0.001** |  | |
| Recipient blood type | **0.139 (0.020)** | | **<0.001** | 0.036 (0.019) | | 0.068 |  | |
| Indication for liver transplantation | **0.195 (0.038)** | | **<0.001** | **0.078 (0.037)** | | **0.034** |  | |
| History of diabetes | **0.058 (0.001)** | | **<0.001** | 0.019 (0.010) | | 0.064 |  | |
| History of renal failure requiring dialysis | **0.004 (0.002)** | | **0.022** | 0.000 (0.001) | | 0.707 |  | |
| Recipient on ventilator support | **0.004 (0.001)** | | **0.018** | 0.001 (0.001) | | 0.352 |  | |
| History of spontaneous bacterial peritonitis | 0.000 (0.003) | | 0.902 | 0.002 (0.003) | | 0.481 |  | |
| History of transjugular portosystemic shunt | 0.005 (0.006) | | 0.528 | 0.001 (0.006) | | 0.925 |  | |
| Portal vein thrombosis | **0.004 (0.007)** | | **0.032** | 0.004 (0.007) | | 0.551 |  | |
| Previous abdominal surgeries | **0.040 (0.011)** | | **<0.001** | 0.021 (0.012) | | 0.071 |  | |
| Recipient on life support | **0.066 (0.004)** | | **0.034** | 0.001 (0.002) | | 0.612 |  | |
| Donor sex | **0.109 (0.011)** | | **<0.001** | **0.013 (0.029)** | | **0.013** |  | |
| Donor body mass index | **0.192 (0.027)** | | **<0.001** | **0.08 (0.014)** | | **<0.001** |  | |
| Donor age | 5.153 (0.374) | | <0.001 | **2.092 (0.380)** | | **<0.001** |  | |
| Year of transplantation | - | | - | **0.373 (0.126)** | | **0.003** |  | |

**Supplementary Table 5B**

| Standardized mean difference and standard error of the difference (SED) before and after propensity score matching between recipients of live donor liver transplantation (LDLT) versus recipients of deceased donor liver transplantation (DDLT) with MELD 16-20. | | | | | | |
| --- | --- | --- | --- | --- | --- | --- |
|  |  |  |  |  |  |  |
| **MELD 16-20** | **Before Propensity Score Matching** | | | **After Propensity Score Matching** | | |
| **Characteristics** | LDLT, n. 1,043 | DDLT, n. 18,798 | **P Value** | LDLT, n. 857 | DDLT, n. 8,162 | **P Value** |
|  | **Standardized Mean Difference (SED)** | |  | **Standardized Mean Difference (SED)** | |  |
| Recipient sex | 0.106 (0.135) | | **<0.001** | **0.039 (0.016)** | | **0.016** |
| Recipient age | 2.019 (0.324) | | **<0.001** | **0.934 (0.355)** | | **0.009** |
| Recipient race/ethnicity | 0.053 (0.016) | | **<0.001** | **0.005 (0.014)** | | 0.701 |
| Recipient body mass index | 0.301 (0.038) | | **<0.001** | **0.280 (0.177)** | | **0.007** |
| Recipient highest degree of education | 0.095 (0.030) | | **<0.001** | 0.037 (0.030) | | 0.224 |
| Recipient functional status | 3.831 (0.650) | | **<0.001** | 1.099 (0.654) | | 0.093 |
| Recipient type of healthcare insurance | 0.156 (0.019) | | **<0.001** | 0.032 (0.019) | | 0.089 |
| Recipient blood type | 0.175 (0.028) | | **<0.001** | 0.047 (0.027) | | 0.080 |
| Indication for liver transplantation | 0.067 (0.204) | | **<0.001** | 0.016 (0.778) | | 0.389 |
| History of diabetes | 0.080 (0.014) | | **<0.001** | 0.021 (0.014) | | 0.126 |
| History of renal failure requiring dialysis | 0.043 (0.006) | | **<0.001** | 0.001 (0.002) | | 0.619 |
| Recipient on ventilator support | 0.007 (0.004) | | 0.087 | 0.000 (0.003) | | 0.906 |
| History of spontaneous bacterial peritonitis | 0.004 (0.006) | | 0.516 | 0.000 (0.007) | | 0.952 |
| History of transjugular portosystemic shunt | 0.041 (0.010) | | **<0.001** | 0.006 (0.009) | | 0.529 |
| Portal vein thrombosis | 0.017 (0.011) | | 0.108 | 0.009 (0.011) | | 0.382 |
| Previous abdominal surgeries | 0.005 (0.016) | | 0.769 | 0.001 (0.016) | | 0.961 |
| Recipient on life support | 0.013 (0.005) | | **0.006** | 0.001 (0.004) | | 0.765 |
| Donor sex | 0.135 (0.016) | | **<0.001** | **0.049 (0.017)** | | **0.003** |
| Donor body mass index | 1.619 (0.208) | | **<0.001** | **0.479 (0.197)** | | **0.016** |
| Donor age | 4.894 (0.208) | | **<0.001** | **1.706 (0.544)** | | **0.002** |
| Year of transplantation | - | | - | **4.623 (0.185)** | | **0.013** |

**Supplementary Table 5C**

| Standardized mean difference and standard error of the difference (SED) before and after propensity score matching between recipients of live donor liver transplantation (LDLT) versus recipients of deceased donor liver transplantation (DDLT) with MELD 21-25. | | | | | | |
| --- | --- | --- | --- | --- | --- | --- |
|  |  |  |  |  |  |  |
| **MELD 21-25** | **Before Propensity Score Matching** | | | **After Propensity Score Matching** | | |
| **Characteristics** | LDLT, n. 470 | DDLT, n. 16,252 | **P Value** | LDLT, n. 386 | DDLT, n. 3,708 | **P Value** |
|  | **Standardized Mean Difference (SED)** | |  | **Standardized Mean Difference (SED)** | |  |
| Recipient sex | **0.081 (0.022)** | | **<0.001** | 0.038 (0.024) | | 0.122 |
| Recipient age | **2.352 (0.500)** | | **<0.001** | 1.014 (0.568) | | 0.074 |
| Recipient race/ethnicity | **0.076 (0.024)** | | **<0.001** | 0.0132 (0.022) | | 0.549 |
| Recipient body mass index | **1.108 (0.277)** | | **<0.001** | 0.303 (0.279) | | 0.279 |
| Recipient highest degree of education | **0.030 (0.045)** | | **<0.001** | 0.018 (0.048) | | 0.709 |
| Recipient functional status | **12.750 (1.061)** | | **<0.001** | **2.025 (1,026)** | | **0.048** |
| Recipient type of healthcare insurance | **0.210 (0.031)** | | **<0.001** | 0.020 (0.028) | | 0.477 |
| Recipient blood type | **0.114 (0.004)** | | **0.002** | 0.043 (0.040) | | 0.282 |
| Indication for liver transplantation | **0.038 (0.090)** | | **<0.001** | 0.009 (0.094) | | 0.921 |
| History of diabetes | **0.102 (0.021)** | | **0.002** | 0.018 (0.020) | | 0.354 |
| History of renal failure requiring dialysis | **0.148 (0.017)** | | **<0.001** | 0.011 (0.008) | | 0.184 |
| Recipient on ventilator support | **0.048 (0.010)** | | **<0.001** | 0.002 (0.002) | | 0.326 |
| History of spontaneous bacterial peritonitis | **0.024 (0.011)** | | **0.024** | 0.007 (0.009) | | 0.474 |
| History of transjugular portosystemic shunt | 0.024 (0.014) | | 0.091 | 0.012 (0.014) | | 0.406 |
| Portal vein thrombosis | **0.058 (0.015)** | | **<0.001** | 0.009 (0.013) | | 0.486 |
| Previous abdominal surgeries | 0.033 (0.023) | | 0.153 | 0.010 (0.025) | | 0.682 |
| Recipient on life support | **0.066 (0.012)** | | **<0.001** | 0.004 (0.006) | | 0.555 |
| Donor sex | **0.129 (0.023)** | | **<0.001** | 0.036 (0.025) | | 0.156 |
| Donor body mass index | **0.624 (0.303)** | | **<0.001** | 0.338 (0.317) | | 0.286 |
| Donor age | **2.739 (0.768)** | | **<0.001** | 0.839 (0.803) | | 0.296 |
| Year of transplantation | - | | - | 0.424 (0.280) | | 0.130 |

**Supplementary Table 5D**

| Standardized mean difference and standard error of the difference (SED) after propensity score matching between recipients of live donor liver transplantation (LDLT) versus recipients of deceased donor liver transplantation (DDLT) with MELD 26-30. | | | | | | |
| --- | --- | --- | --- | --- | --- | --- |
|  |  |  |  |  |  |  |
| **MELD 26-30** | **Before Propensity Score Matching** | | | **After Propensity Score Matching** | | |
| **Characteristics** | LDLT, n. 134 | DDLT, n. 12,974 | **P Value** | LDLT, n. 107 | DDLT, n. 875 | **P Value** |
|  | **Standardized Mean Difference (SED)** | |  | **Standardized Mean Difference (SED)** | |  |
| Recipient sex | 0.030 (0.042) | | 0.475 | 0.065 (0.050) | | 0.194 |
| Recipient age | 0.866 (0.970) | | 0.372 | 0.017 (1.166) | | 0.988 |
| Recipient race/ethnicity | **0.154 (0.049)** | | **0.002** | 0.033 (0.373) | | 0.371 |
| Recipient body mass index | 0.592 (0.541) | | 0.907 | 0.534 (0.596) | | 0.371 |
| Recipient highest degree of education | **0.031 (0.083)** | | **0.008** | 0.073 (0.101) | | 0.470 |
| Recipient functional status | **20.433 (2.067)** | | **<0.001** | 1.736 (2.322) | | 0.455 |
| Recipient type of healthcare insurance | **0.219 (0.057)** | | **<0.001** | 0.081 (0.057) | | 0.156 |
| Recipient blood type | **0.211 (0.071)** | | **0.006** | 0.022 (0.072) | | 0.757 |
| Indication for liver transplantation | **0.878 (0.027)** | | **<0.001** | 0.110 (0.191) | | 0.566 |
| History of diabetes | **0.088 (0.037)** | | **0.006** | 0.011 (0.042) | | 0.792 |
| History of renal failure requiring dialysis | **0.219 (0.038)** | | **<0.001** | 0.028 (0.027) | | 0.289 |
| Recipient on ventilator support | **0.087 (0.026)** | | **<0.001** | 0.013 (0.018) | | 0.459 |
| History of spontaneous bacterial peritonitis | **0.046 (0.022)** | | **0.036** | 0.006 (0.019) | | 0.747 |
| History of transjugular portosystemic shunt | **0.049 (0.024)** | | **0.045** | 0.007 (0.023) | | 0.777 |
| Portal vein thrombosis | **0.065 (0.027)** | | **0.017** | 0.011 (0.026) | | 0.673 |
| Previous abdominal surgeries | 0.015 (0.043) | | 0.721 | 0.036 (0.050) | | 0.473 |
| Recipient on life support | **0.141 (0.031)** | | **<0.001** | 0.013 (0.018) | | 0.459 |
| Donor sex | **0.1148 (0.043)** | | **<0.001** | 0.004 (0.051) | | 0.934 |
| Donor body mass index | 0.880 (0.523) | | 0.198 | 0.137 (0.585) | | 0.815 |
| Donor age | 2.344 (1.365) | | 0.086 | 1.510 (1.595) | | 0.344 |
| Year of transplantation | - | | - | 0.666 (0.549) | | 0.226 |

**Supplementary Table 5E**

| Standardized mean difference and standard error of the difference (SED) after propensity score matching between recipients of live donor liver transplantation (LDLT) versus recipients of deceased donor liver transplantation (DDLT) with MELD>30. | | | | | | | | |
| --- | --- | --- | --- | --- | --- | --- | --- | --- |
|  |  |  |  |  |  |  |  |  |
| **MELD > 30** | **Before Propensity Score Matching** | | | **After Propensity Score Matching** | | | |  |
| **Characteristics** | LDLT, n. 466 | DDLT, n. 20,191 | **P Value** | LDLT, n. 466 | DDLT, n. 3,240 | **P Value** |  |  |
|  | **Standardized Mean Difference (SED)** | |  | **Standardized Mean Difference (SED)** | |  |  |  |
| Recipient sex | 0.206 (0.072) | | **0.004** | 0.112 (0.084) | | 0.182 |  |  |
| Recipient age | 0.529 (1.735) | | 0.760 | 0.010 (2.047) | | 0.996 |  |  |
| Recipient race/ethnicity | 0.214 (0.092) | | **0.044** | 0.000 (0.042) | | 0.988 |  |  |
| Recipient body mass index | 1.205 (0.965) | | 0.090 | 0.312 (0.984) | | 0.751 |  |  |
| Recipient highest degree of education | 0.018 (0.156) | | 0.436 | 0.088 (0.161) | | 0.586 |  |  |
| Recipient functional status | 17.487 (3.298) | | **<0.001** | 5.506 (4.181) | | 0.189 |  |  |
| Recipient type of healthcare insurance | 0.041 (0.097) | | 0.436 | 0.026 (0.107) | | 0.811 |  |  |
| Recipient blood type | 0.121 (0.121) | | 0.627 | 0.020 (0.123) | | 0.869 |  |  |
| Indication for liver transplantation | 0.016 (0.347) | | **0.014** | 0.051 (0.359) | | 0.887 |  |  |
| History of diabetes | 0.025 (0.062) | | 0.690 | 0.003 (0.703) | | 0.966 |  |  |
| History of renal failure requiring dialysis | 0.243 (0.070) | | **<0.001** | 0.051 (0.059) | | 0.387 |  |  |
| Recipient on ventilator support | 0.054 (0.052) | | 0.312 | 0.008 (0.052) | | 0.877 |  |  |
| History of spontaneous bacterial peritonitis | 0.030 (0.039) | | 0.446 | 0.002 (0.037) | | 0.947 |  |  |
| History of transjugular portosystemic shunt | 0.056 (0.040) | | 0.163 | 0.006 (0.029) | | 0.839 |  |  |
| Portal vein thrombosis | 0.032 (0.040) | | 0.427 | 0.007 (0.043) | | 0.867 |  |  |
| Previous abdominal surgeries | 0.011 (0.073) | | 0.879 | 0.002 (0.082) | | 0.982 |  |  |
| Recipient on life support | 0.0133 (0.062) | | **0.032** | 0.008 (0.052) | | 0.877 |  |  |
| Donor sex | 0.206 (0.074) | | **0.005** | 0.050 (0.084) | | 0.552 |  |  |
| Donor body mass index | 0.859 (0.849) | | **0.012** | 0.025 (0.873) | | 0.976 |  |  |
| Donor age | 1.238 (2.269) | | 0.585 | 2.084 (2.479) | | 0.290 |  |  |
| Year of transplantation | - | | - | 0.240 (0.906) | | 0.979 |  |  |


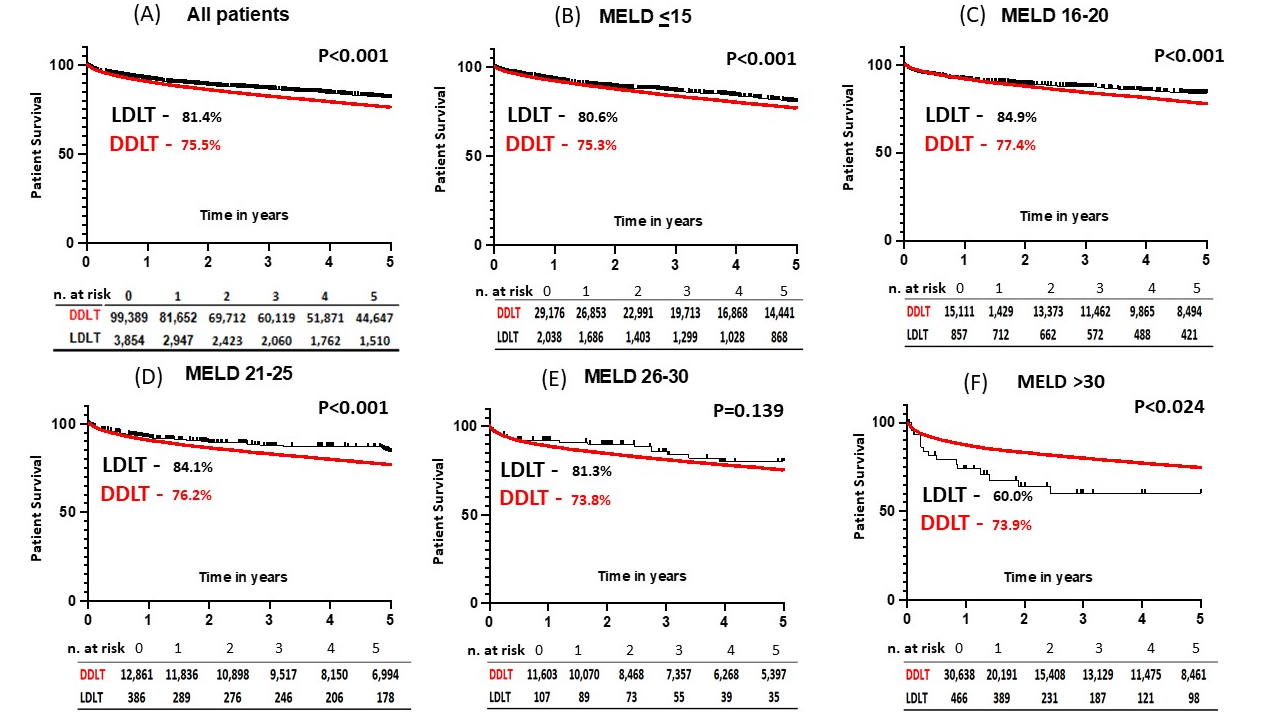


S**upplementary Figure 1.** Unadjusted patient survival functions before propensity score matching between recipients of live donor liver transplantation (LDLT) and recipients of deceased donor liver transplantation (DDLT). Panel A compares the survival between the two groups of patients irrespective of the model for end stage liver disease (MELD) at the time of surgery. Panel B-F illustrates the survival of LDLT versus DDLT recipients stratified by their MELD scores at the time of liver transplantation.
